# Combining Dense Longitudinal Records from Robotic Milking with Dense On-Farm Meteorological Data to Assess Heat Stress Effects in Dairy Cows

**DOI:** 10.3390/ani16172671

**Published:** 2026-08-25

**Authors:** Elena Frenken, Kerstin Brügemann, Sven König

**Affiliations:** 1 Institute of Animal Breeding and Genetics, Justus-Liebig-University of Gießen, Ludwigstr. 21b, 35390 Giessen, Germany; kerstin.bruegemann@agrar.uni-giessen.de (K.B.); sven.koenig@agrar.uni-giessen.de (S.K.); 2 Association for Bioeconomy Research, Adenauerallee 174, 53113 Bonn, Germany

**Keywords:** heat stress, dairy cows, automatic milking systems, temperature–humidity index, milking robot traits, longitudinal data structure, on-farm meteorological data

## Abstract

Climate change is increasing the frequency of heat stress events in dairy farming, adversely affecting milk production, milk composition, cow behavior, and animal health. However, many previous studies relied on distant weather-station data and low-frequency milk recording systems, limiting the assessment of short-term and delayed heat stress responses. Therefore, the aim of this study was to combine dense longitudinal data from automatic milking systems (AMS) with continuously recorded on-farm meteorological measurements to investigate the immediate and lagged effects of heat stress on Holstein dairy cows. The study included 386,587 AMS visit records from 790 cows on three commercial dairy farms in Germany, corresponding to up to 127,310 cow-day records collected between August 2022 and August 2025. Temperature–humidity index (THI) values were calculated based on dense on-farm temperature and relative humidity records and were evaluated for multiple lag periods prior to AMS recordings. Linear mixed models were applied to infer the effects of THI on production, physiological, behavioral, and milking process traits. Increasing THI was associated with reduced daily milk yield, altered milk fat and protein percentages, decreased AMS visit frequency, prolonged milking intervals, and increased milk temperature. For contemporaneous THI, an increase from THI 50 to THI 70 corresponded to model-estimated declines of −0.86 kg in daily milk yield, −0.20% in milk fat content and −0.06% in milk protein content, −0.12 daily AMS visits, and +1.14 °C in milk temperature. The strongest associations were generally observed for prompt and short-term lagged THI windows. In contrast, longer lag periods were associated with weaker and less distinct trait responses. Rather than merely confirming the established decline in milk yield under heat stress, the integrated and temporally resolved analysis revealed trait-specific response patterns across production, behavioral, physiological, health-related, and milking-process traits. In particular, milk temperature and voluntary AMS attendance showed pronounced associations with contemporaneous and short-term THI, demonstrating the value of combining AMS-derived phenotypes with high-resolution on-farm climate data for heat stress monitoring.

## Simple Summary

Automatic milking systems (AMS) generate large amounts of high-frequency data on the milk production, milking behavior, and physiological responses of dairy cows. At the same time, climate change is increasing the frequency and intensity of heat stress events, even in temperate regions such as Germany. Heat stress adversely affects milk production, milk composition, cow behavior, and animal health. However, many previous studies relied on distant weather-station data and low-frequency milk recording systems, limiting the assessment of short-term and delayed heat stress responses. In this study, dense AMS data from three commercial dairy farms were combined with continuously recorded on-farm meteorological measurements to investigate the immediate and delayed effects of heat stress on dairy cows. Different temperature–humidity index (THI) periods prior to each AMS recording were evaluated to assess temporal heat stress effects. Increasing THI was associated with lower milk yield, altered milk composition, reduced AMS visit frequency, prolonged milking intervals, and increased milk temperature. The strongest responses in all trait categories were observed for prompt and short-term heat stress exposure. These findings demonstrate the potential of combining AMS-derived phenotypes with on-farm climate data for improved heat stress monitoring and management in dairy farming.

## 1. Introduction

Climate change and the associated weather extremes have direct and indirect impacts on livestock production. Heat stress is one of the most important climatic stressors affecting dairy cow health, welfare and performance [1]. As cows struggle to maintain thermal balance, a cascade of physiological adjustments is triggered: they increase respiratory rate and sweating to dissipate heat [2], but if body heat excess cannot be shed, internal temperature rises. Such a physiological response, i.e., hyperthermia, can be detected as an increase in milk temperature (closely correlated with core body temperature) [2]. Endocrine changes and reduced feed intake under heat stress further disrupt metabolism [2]. Consequently, heat-stressed cows exhibit reduced milk yield and altered milk composition, along with impaired fertility and higher disease susceptibility [3].

To quantify environmental heat load, the Temperature–Humidity Index (THI) is commonly used. The THI combines ambient temperature and relative humidity into a single metric reflecting thermal conditions experienced by the animal [3]. Although threshold values vary depending on climate, production level, and management conditions, THI values close to 68 are commonly considered as indicators for the onset of heat stress in dairy cattle [1,4].

Most classical heat-stress research in dairy cattle has been based on external meteorological data and conventional low-frequency phenotypes. For example, Brügemann et al. analyzed 1,095,980 first-lactation test-day records for protein yield from 154,880 Holstein cows kept on 196 German farms, and merged these data with public weather-station measurements, implying distances of up to 50 km between farm and station [5]. Although such studies established that rising THI is associated with lower performance even under temperate conditions, official weather-station data may not adequately reflect the barn microclimate, and conventional test-day records may miss short-lived heat events and trait-specific temporal dynamics.

A further methodological development was therefore the use of on-farm climatic measurements. Gernand et al. combined on-farm temperature and relative humidity records with conventional longitudinal cow traits from 22,212 Holstein cows in 15 large-scale dairy herds and quantified the THI effects on productivity, female fertility, and health [6]. Their approach linked barn-level THI to conventional monthly test-day records and demonstrated that both same-day THI and THI from the previous week can be relevant, depending on the response trait of interest. However, the recording frequency still limited the assessment of the dense phenotypes in association analyses, but longitudinal records are now widely accessible from robotic milking systems.

The increasing availability of automatic milking systems (AMS) enables a more detailed assessment of cow responses to heat stress. In contrast to conventional monthly test-day recording, AMS generate dense longitudinal datasets with multiple observations per cow and day, including detailed information on milk production, milk composition, cow traffic, milking behavior, and physiological parameters [3]. Furthermore, modern AMS record milk temperature continuously during milking. Previous studies demonstrated that heat stress increases body temperature and impairs thermoregulation in dairy cows, which may also be reflected in elevated milk temperature measured during AMS milking [7,8]. In contrast to indirect production responses such as declining milk yield, milk temperature may reflect more immediate physiological reactions to thermal stress.

Combining dense AMS-derived phenotypes with simultaneously recorded on-farm climatic data allows the assessment of immediate, delayed, and cumulative heat-stress responses. This is particularly relevant because the timing of heat-stress responses may differ among traits. For example, Hagiya et al. reported lagged responses of milk yield and somatic cell score to heat stress exposure in Holstein cows [9]. Likewise, Kipp et al. demonstrated across-generation effects of maternal heat stress during late gestation on subsequent production, fertility, and longevity traits in dairy cattle, emphasizing that thermal stress can induce long-lasting biological consequences beyond immediate production responses [10]. However, high-frequency AMS phenotypes and dense on-farm climatic measurements have rarely been combined to characterize trait-specific temporal responses under commercial farming conditions.

Given this research gap, the objective of the present study was to combine dense longitudinal AMS data reflecting production, behavior, physiology, udder health, and the technical milking process with simultaneously recorded barn-level meteorological measurements. Contemporaneous and time-lagged THI effects were evaluated across multiple exposure windows to characterize trait-specific differences in the timing and shape of heat-load associations under commercial housing conditions. This integrated approach was intended to assess the potential of routinely recorded AMS traits for temporally resolved heat-stress monitoring beyond conventional milk-production indicators.

## 2. Materials and Methods

### 2.1. AMS Herds

This study was conducted in the period from August 2022 to August 2025 using longitudinal milking robot data from three commercial dairy farms located in the German federal states of North Rhine-Westphalia. All farms kept Holstein dairy cows under practical production conditions and were equipped with Lely Astronaut automatic milking systems (Lely Industries N.V., Maassluis, The Netherlands). The three farms kept 175, 235, and 90 milking cows, respectively. The first farm operated three Lely Astronaut A4 units, the second farm four Lely Astronaut A4 units, and the third farm two Lely Astronaut A5 units. All farms applied free cow traffic, allowing cows voluntary access to the AMS.

### 2.2. Meteorological Data and Meteorological Data Preparation

On-farm meteorological conditions were continuously recorded inside the barns using permanently installed climate data loggers (Tinytag Plus 2 USB Data Logger TGP-4500; Gemini Data Loggers Ltd., Chichester, UK). According to the manufacturer’s protocol, the logger has a temperature measurement range from −25 °C to +85 °C and a reading resolution of 0.01 °C or better. Temperature accuracy is temperature-dependent and corresponds to approximately ±0.4 °C to ±0.6 °C across the temperature range observed in the present study. Relative humidity is measured over a range from 0% to 100%, with a manufacturer-specified accuracy of ±3.0% RH at 25 °C and a reading resolution better than 0.3% RH [11]. Ambient temperature (Temp, °C) and relative humidity (RH, %) were measured in hourly intervals throughout the entire recording period. The sensors were positioned within the barn environment, specifically in the feeding alley, to capture the climatic conditions directly experienced by the cows.

Meteorological data were exported in XLSX format and processed using SAS 9.4M8 via SAS Studio 3.82, Enterprise Edition (SAS Institute Inc., Cary, NC, USA). Prior to THI calculation, the records were screened for plausibility. Relative-humidity values below 20% or above 99% were replaced by the most recent preceding valid measurement. All recorded temperature values, ranging from −10 °C to 39 °C, were retained because they were considered plausible for the investigated barn conditions and recording period. Daily THI was calculated according to the equation of the National Research Council [12] based on the average daily measurements for Temp and RH:

THI = (1.8 × Temp + 32) − (0.55 − 0.0055 × RH) × (1.8 × Temp − 26).

**Figure 1:**
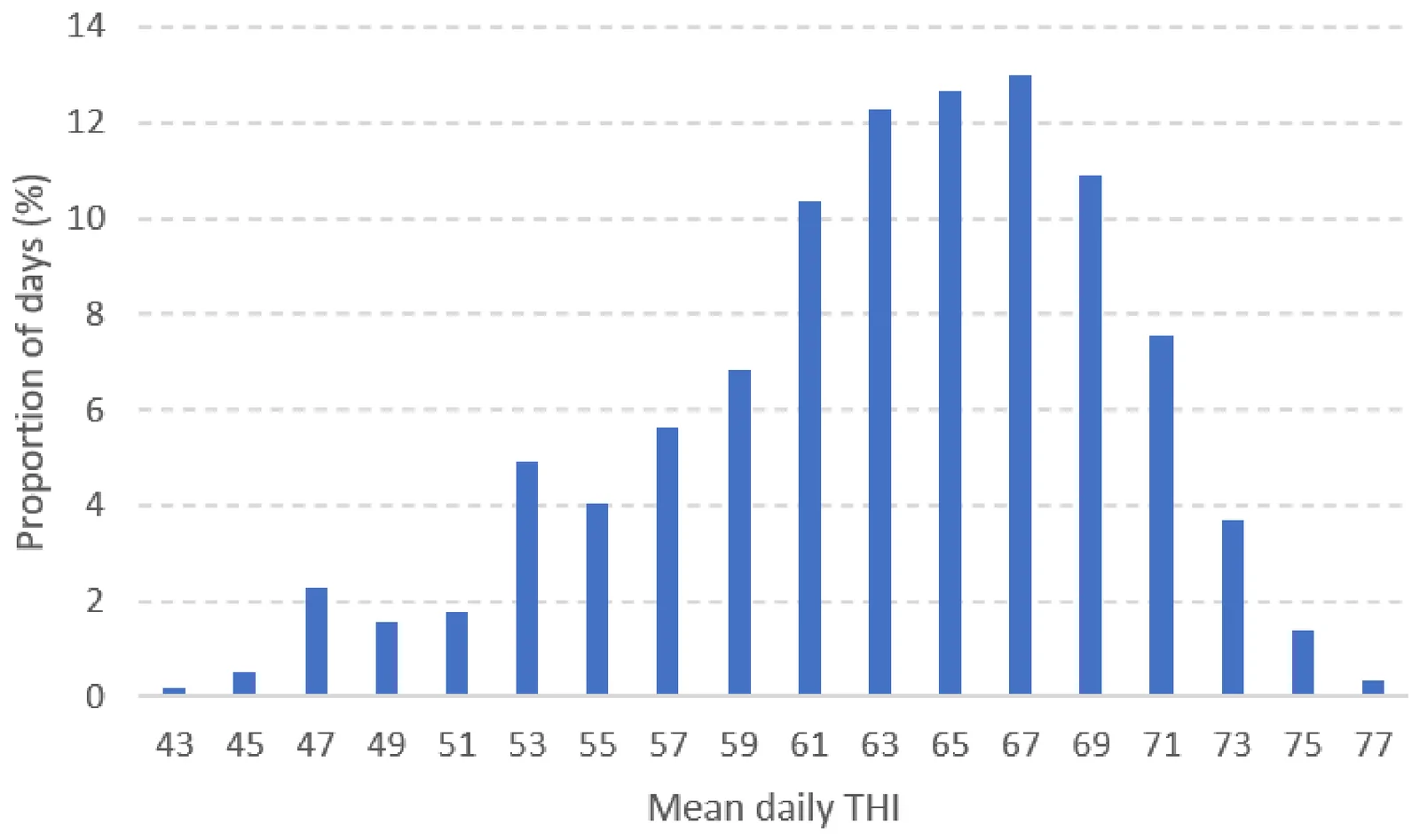
Distribution of mean daily temperature–humidity index recorded inside the study barns between August 2022 and August 2025.

To evaluate both immediate and delayed heat stress effects, several THI metrics representing different exposure periods before the respective AMS observation, were derived. In addition to the average THI of the recording day (THI_0_), average THI values for cumulative lag periods before cow trait recording were calculated. These included THI values averaged across the preceding 1–3 days (THI_1–3_), 1–7 days (THI_1–7_), 8–14 days (THI_8–14_), 15–21 days (THI_15–21_), and 22–28 days (THI_22–28_) before the respective AMS recording. The distribution of THI records for the THI_0_ recording period is shown in Figure 1.

### 2.3. AMS Cow Traits

#### 2.3.1. Milking Robot Production Traits

Cow production traits included daily milk yield (DMY), daily milk fat percentage (F%), and daily milk protein percentage (P%) generated in the AMS. The content traits F% and P% were recorded via inline milk analysis sensors integrated into the milking robot, based on near-infrared spectroscopy and calibrated reference measurements, as described by Kawasaki et al. [13].

#### 2.3.2. Milking Robot Behavior Traits

Regarding behavior trait recording, it was imperative for the present study to select herds with voluntary cow traffic as outlined by König et al. [14]. In such milking robot environments, milking-robot-related behavior traits reflect the natural cow behavior by limiting environmental effects due to the husbandry system. Behavioral traits included number of daily AMS visits (NVISITS), milking interval (MINT), scan time (STIME), and connecting time (CTIME). NVISITS represented the daily number of successful voluntary visits to the AMS, whereas MINT described the time interval in hours between two consecutive milkings. STIME referred to the duration in seconds required for teat detection and udder scanning before attachment of the milking unit, whereas CTIME in seconds described the time needed for successful attachment of the milking device. To ensure data quality, biologically implausible and technically invalid records were removed prior to the statistical analyses. Specifically, STIME values of 0 s or >60 s and CTIME values of 0 s or >120 s were excluded. In addition, records with more than 10 udder scans were discarded, and MTEMP values <36 °C or >42 °C were considered biologically implausible and treated as missing.

The initial dataset comprised 386,587 AMS records from 790 cows. After aggregation of the visit-level records, 127,310 cow-day records were available for daily milk yield and 127,293 cow-day records for the number of daily AMS visits. Because quality-control criteria, missing values, and the availability of the respective THI exposure periods were considered separately for each response trait, the number of observations included in the statistical models varied among traits and exposure windows.

#### 2.3.3. AMS Physiological and Health Indicator Traits

Physiological and health-related traits included milk temperature (MTEMP) and somatic cell score (SCS). Somatic cell count was automatically measured during milking using AMS-integrated cell count sensors based on optical and/or fluorescence detection principles, allowing repeated high-frequency udder health monitoring under practical farm conditions [15]. Somatic cell count (SCC) was transformed to SCS using the commonly applied logarithmic transformation according to Ali and Shook [16] and Wiggans and Shook [17]:

*SCS* = *log*_2_ (*SCC*/100,000) + 3

where *SCC* is expressed in cells/mL.

Cows showing clinical signs of disease were managed according to the routine herd-management procedures of the respective commercial farms and were therefore not included in the analyzed records during periods of clinical illness. No additional exclusion criterion based on SCC or SCS thresholds was applied for subclinical mastitis, as SCS itself was included as a response trait to characterize variation in udder health.

Milk temperature (MTEMP) was recorded automatically during each milking event using temperature sensors integrated into the AMS. During milking, milk temperature was continuously measured within the milking system during milk flow, and the maximum milk temperature per milking event was stored by the AMS [18].

Descriptive statistics for all cow traits from the categories AMS production, AMS behavior and AMS physiology are shown in Table 1.

#### 2.3.4. AMS Data Extraction and Linkage with Meteorological Data

Relevant variables were extracted from the Lely AMS backup files of the participating farms. Each milking record was assigned to its calendar date based on the AMS timestamp and linked, within farm, to the mean barn-level THI recorded on the corresponding day (THI_0_). The record was additionally linked to the pre-calculated mean THI values for the defined preceding periods THI_1–3_, THI_1–7_, THI_8–14_, THI_15–21_, and THI_22–28_.

### 2.4. Statistical Association Analyses

Linear mixed models were applied to infer the effects of THI from the prompt and time-lagged periods on milking robot traits. In this regard, the SAS procedure MIXED (Version 9.4M8, 2026; SAS Institute, Cary, NC, USA) was applied. The full statistical model was defined as follows:

$$\begin{array}{c} y_{ijklmnop}={µ+\;Lac}_{i}+{HYS}_{j}+{Robot}_{k}+{Groupsize}_{l}+{Daytime}_{m}+\\ \sum_{p=0}^{4}{\alpha_{n}Z}_{n}(DIM)+\sum_{q=0}^{4}\beta_{o}Z_{o}(THI)+e_{ijklmnop} \end{array}$$

where *y_ijklmnop_* is the daily record for DMY and NVISITS, or single records from specific AMS visits for F%, P%, SCS, MTEMP, MINT, STIME and CTIME; *µ* = overall mean; *Lac_i_* = fixed effect for the *i*-th lactation number (*i* = 1, 2, 3, 4, or >4); *HYS_j_* = fixed effect for the *j*-th herd-year-season class; *Robot_k_* = fixed effect for the *k*-th milking robot of the herd; *Groupsize_l_* = fixed effect for the *l*-th group for the number of cows per milking robot (l = <40 cows, 40–55 cows or ≥55 cows); *Daytime_m_* = fixed effect for the time of the day (*m* = 20.00 h to 4.59 h, 5.00 to 12.59 h, or 13.00 to 19.59 h); *α_n_* = fixed regression coefficient for the *n*-th days in milk (DIM); *β_o_* = random regression coefficient of the *p*-th cow by *o*-th THI; *Zn* and *Zo* = vector of Legendre polynomials (order 4) describing the shape of the fixed lactation curve and random THI curve, respectively; and $e_{ijklmnop}$ = random residual error term. A variance-components covariance structure (type = vc) was specified for the random regression coefficients, assuming zero covariances among the random effects. This parsimonious structure was selected to provide stable estimation of the population-level THI response curves, as modeling more complex animal-specific covariance patterns was not the primary objective of this study. 

For the observations on a per day basis (DMY, NVISITS), the fixed effect *Daytime* was excluded from the statistical model. Series of consecutive runs were performed for all combinations of traits and THI time windows.

Wald-type tests were used to identify significant fixed effects on AMS traits (type III tests of fixed effects). Least squares means (LSMeans) for all levels along the continuous THI gradient were computed by applying the “at-statement” for serial data as implemented in Proc Mixed of SAS and as described by Littel et al. for repeated measures analyses [19]. Representative detailed SAS outputs for milk protein percentage (P%), milking interval (MINT), and milk temperature (MTEMP) for the THI_1–3_ exposure window are provided in Supplementary File S1. These traits represent the production, behavioral, and physiological trait categories, respectively.

## 3. Results

The distribution of mean daily THI values across the recording period is presented in Figure 1. The central THI range was represented by a larger number of recording days, whereas fewer days occurred at some portions of the THI extremes. Estimates at less frequently represented THI values should therefore be interpreted with corresponding caution. Representative detailed model outputs for selected production, behavioral, and physiological traits are provided in Supplementary File S1. Analyses of variance indicated mostly non-significant effects (*p* > 0.05) for the time-lagged period THI_22–28_ on all AMS response traits. Consequently, the THI_22–28_ time window was not included in the LSMeans figures presented below, and the focus of the subsequent results is placed on the prompt and short-term lagged periods (THI_0_ to THI_15–21_).

### 3.1. Milk Production Traits

**Figure 2:**
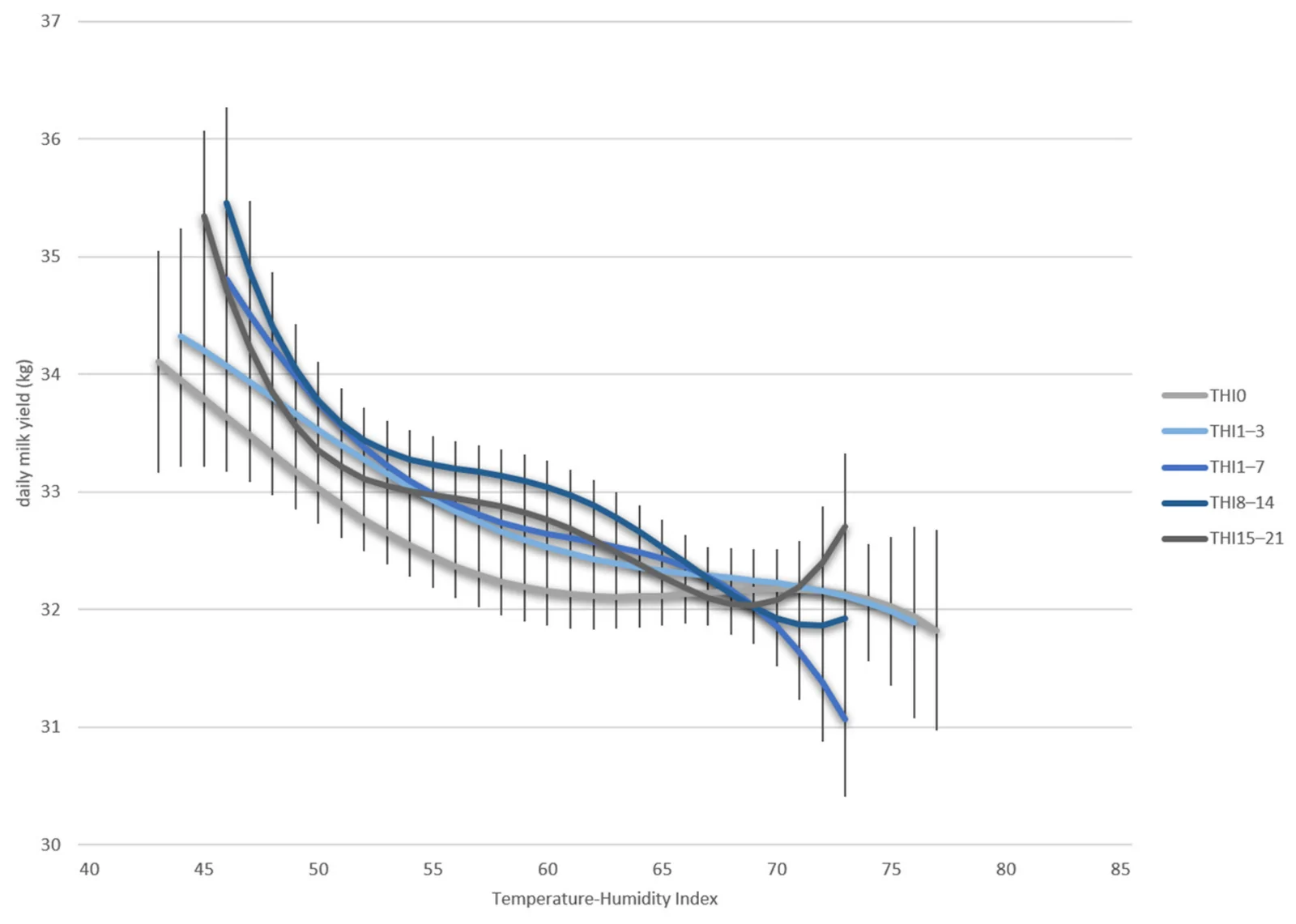
Least squares means for daily milk yield (DMY, in kg), with corresponding SEs (vertical bars) by THI from different recording periods (THI_0_, THI_1–3_, THI_1–7_, THI_8–14_, THI_15–21_) before trait recording.

Least squares means for DMY generally declined with increasing THI, indicating a clear negative association between heat load and milk production (Figure 2). Across all THI windows investigated, DMY decreased consistently as THI increased. The strongest differentiation in DMY was observed across the THI levels of the contemporaneous and short-term lagged THI windows (THI_0_, THI_1–3_, and THI_1–7_), which showed pronounced declines in milk yield with increasing THI. The response was characterized by a steep reduction at low to intermediate THI, followed by a more gradual decline at higher THI. In contrast, the longer lag periods (THI_8–14_ and particularly THI_15–21_) exhibited flatter response curves and less distinct differences across the THI range. At elevated THI levels, the differences in least squares means for DMY between the different THI windows became progressively smaller, indicating that milk yield was consistently reduced under conditions of high thermal load regardless of the timing of heat stress exposure. Overall, the results suggest that milk yield responded primarily to contemporaneous and recent heat stress exposure, whereas associations with more distant heat stress periods were less pronounced.

**Figure 3:**
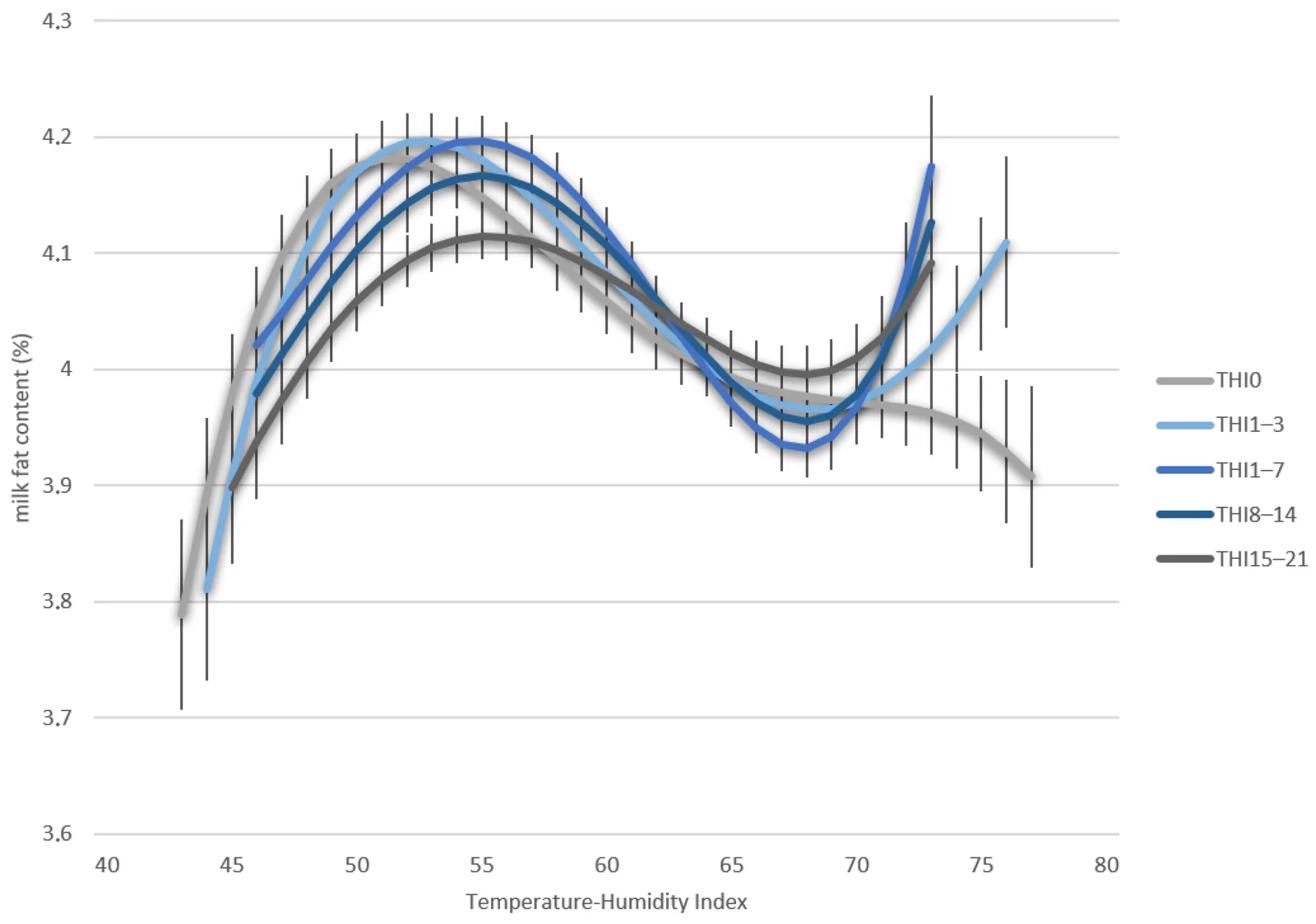
Least squares means for milk fat content (F%), with corresponding SEs (vertical bars) by THI from different recording periods (THI_0_, THI_1–3_, THI_1–7_, THI_8–14_, THI_15–21_) before trait recording.

Milk fat percentage showed a non-linear association with increasing THI across all investigated time windows (Figure 3). In general, the LSMeans for F% increased from lower THI towards intermediate THI classes and subsequently declined again at higher THI. For the prompt THI time window (THI_0_), F% increased from approximately 3.7–3.9% at low THI to maximum levels of 4.2% at intermediate THI ranges around 50–55, followed by a decline towards higher THI. Such an inverted U-shaped curve pattern was consistently observed across all lagged THI windows. The strongest curvature was evident for THI_1–7_ and THI_8–14_, where LSMeans for F% exceeded 4.2% at intermediate THI levels before declining to approximately 4.0% at THI beyond 65. In contrast, the THI_15–21_ modelling approach implied a flatter response pattern characterized by a more gradual F% increase across the THI range, and a less pronounced F% decline at higher THI. Overall, the results indicate that F% responded non-linearly to increasing THI, with the highest LSMeans observed at moderate THI, whereas both low and high THI conditions were associated with comparatively lower fat percentages.

**Figure 4:**
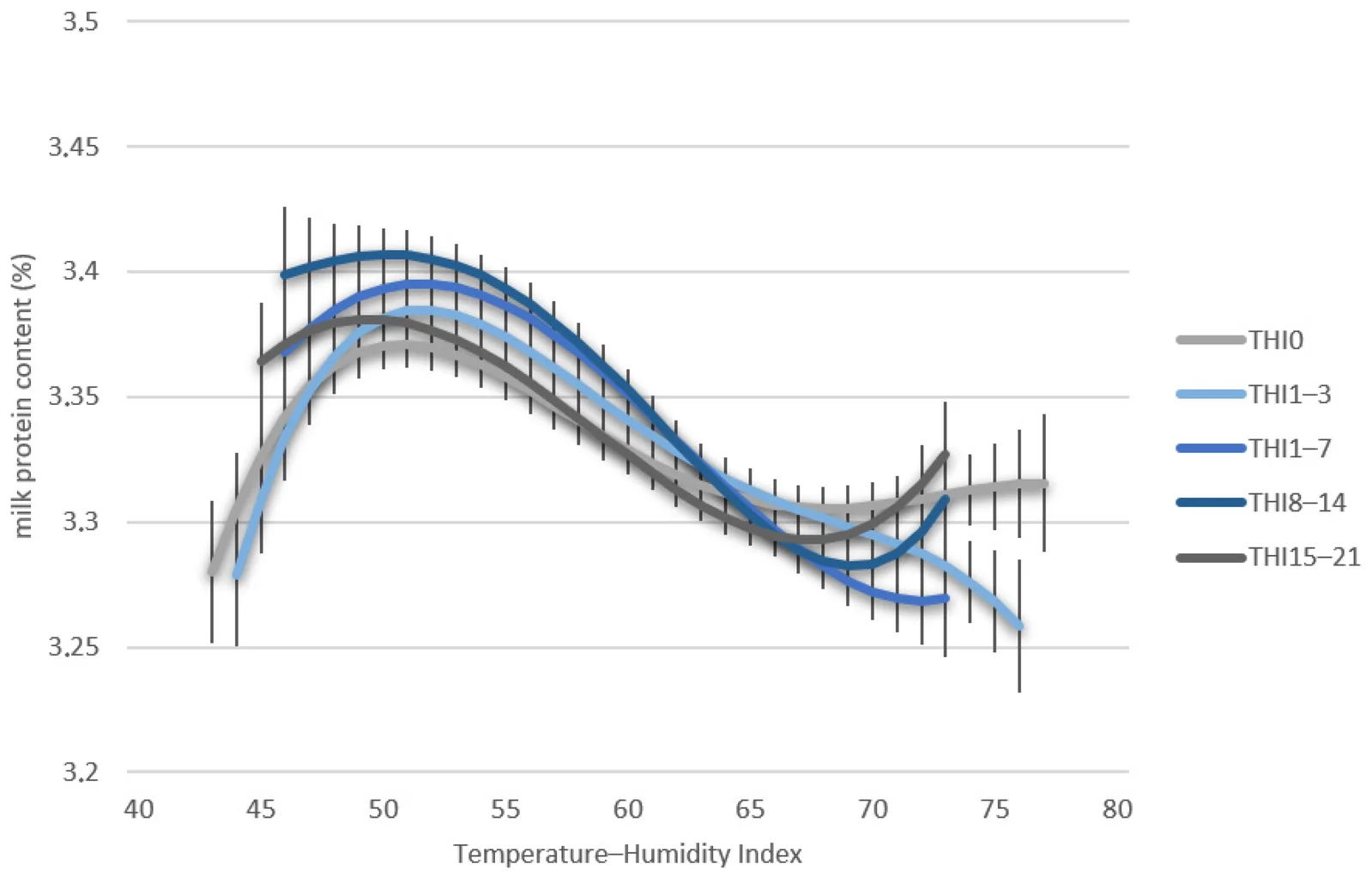
Least squares means for milk protein content (P%), with corresponding SEs (vertical bars) by THI from different recording periods (THI_0_, THI_1–3_, THI_1–7_, THI_8–14_, THI_15–21_) before trait recording.

Milk protein percentage showed a predominantly declining response at higher THI values, particularly for the lagged exposure windows (Figure 4). Compared with F%, the LSMeans for P% showed a more consistent declining response pattern at higher THI levels, particularly for the lagged THI windows. For the prompt THI window (THI_0_), P% initially increased slightly from approximately 3.29% at lower THI to maximum levels around 3.37% at intermediate THI ranges between 50 and 55. Beyond this range, LSMeans for P% gradually declined towards a higher THI. The decline was more pronounced for the lagged THI windows THI_1–3_ and THI_1–7_, where P% decreased markedly at a THI above 55 and reached minimum values at a THI exceeding 70. The strongest P% decline was observed for THI_8–14_, where LSMeans for P% decreased to approximately 3.25% at the highest THI. In contrast, the THI_15–21_ modelling approach implied a comparatively flat curve pattern with a less pronounced reduction across the THI range. Overall, the strongest associations between THI and P% were observed for lagged THI windows representing heat stress exposure within one to two weeks prior to trait recording, whereas a prompt THI induced weaker and more gradual P% responses.

**Figure 5:**
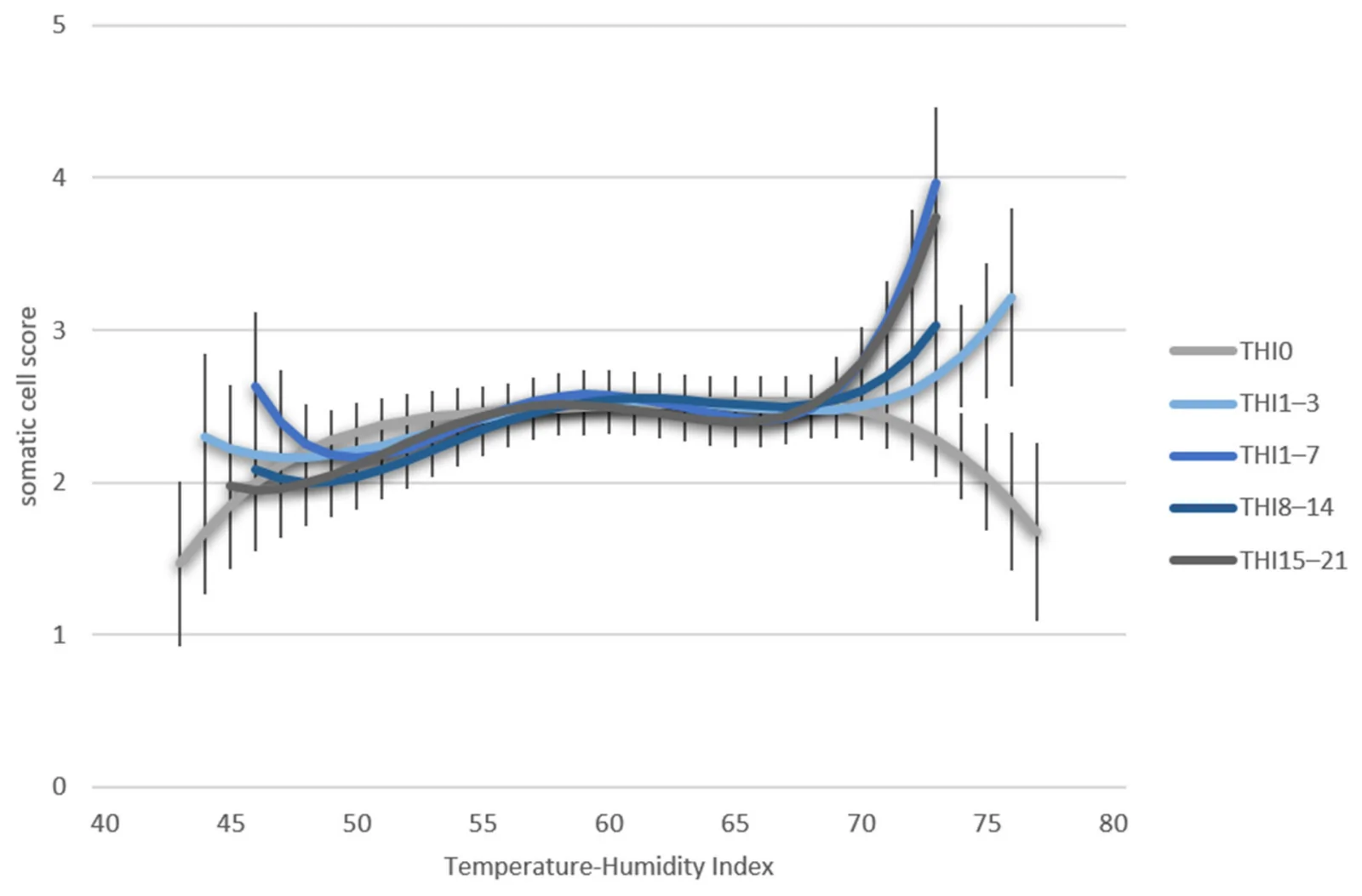
Least squares means for somatic cell score (SCS) with corresponding SEs (vertical bars) by THI from different recording periods (THI_0_, THI_1–3_, THI_1–7_, THI_8–14_, THI_15–21_) before trait recording.

Somatic cell score tended to increase at higher THI values, although the shape and magnitude of the association reflected though the LSMeans pattern varied considerably among the exposure windows (Figure 5). The modelling approach for prompt THI effects (THI_0_) and the short-term lagged time windows (THI_1–3_ and THI_1–7_) generally indicated higher SCS values at elevated THI levels, suggesting a tendency towards impaired udder health under increasing heat load. However, the shape of the response curves varied across the THI windows, and fluctuations became more pronounced, especially at higher THI. Intermediate and longer lagged periods (THI_8–14_ and THI_15–21_) showed less distinct SCS responses and only weak differentiations across THI. Overall, the LSMeans for SCS indicate a tendency for increased SCS under higher thermal load, although the relationship was more variable and less pronounced than for production and behavioral traits.

### 3.2. AMS Behavior Traits

**Figure 6:**
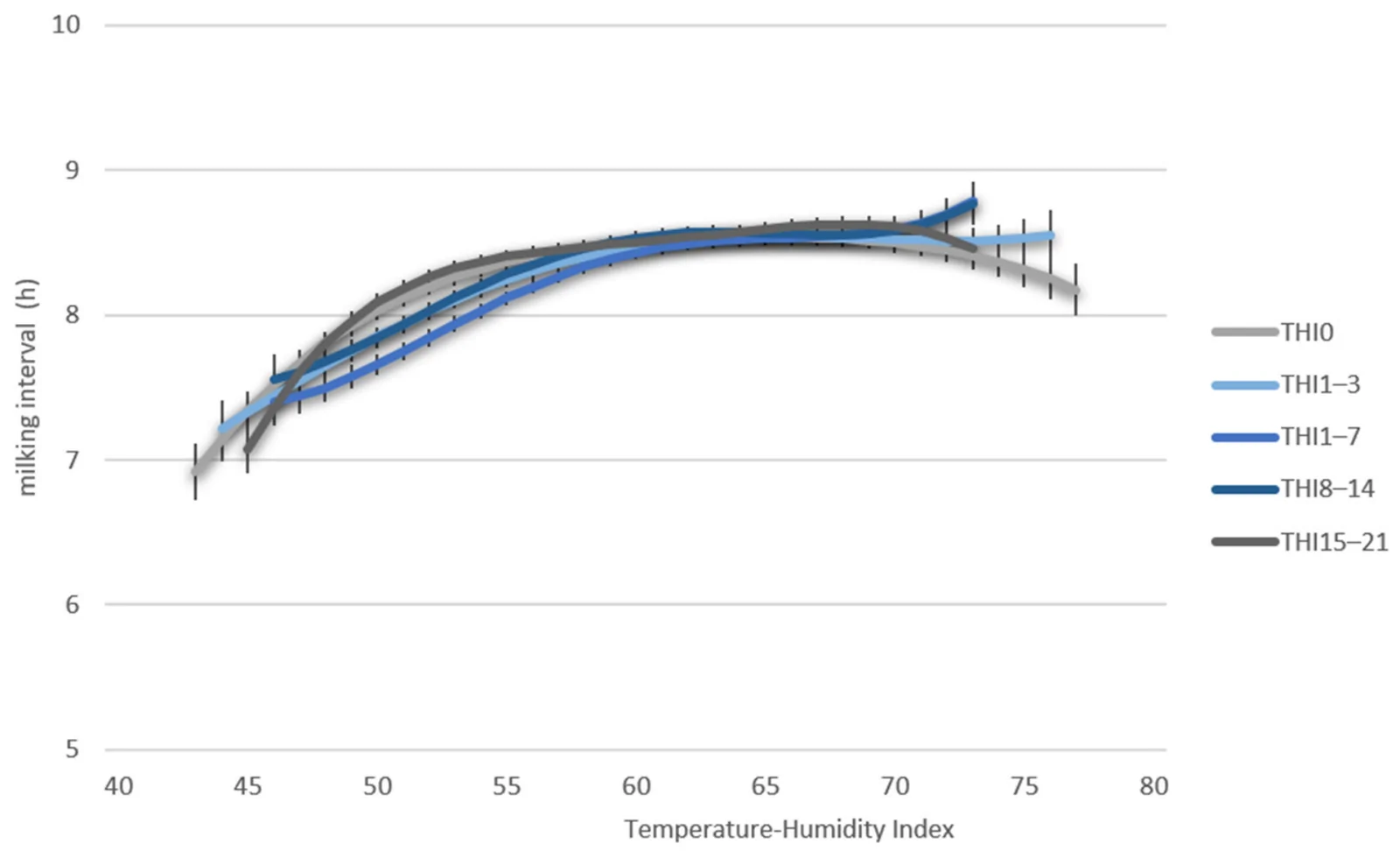
Least squares means for milking interval (MINT) (h), with corresponding SEs (vertical bars) by THI from different recording periods (THI_0_, THI_1–3_, THI_1–7_, THI_8–14_, THI_15–21_) before trait recording.

Least squares means for MINT displayed non-linear associations with increasing THI across all investigated time windows (Figure 6). In general, MINT increased from lower towards intermediate THI levels and subsequently declined slightly again at higher THI. With regard to the lower THI range from THI 40 to THI 50, LSMeans for MINT increased markedly from around 7.0 h to values exceeding 8.0 h. The highest LSMeans for MINT were observed at an intermediate THI between THI 55 and THI 65, i.e., milking intervals ranging from 8.5 to 9.0 h, depending on the investigated THI window. At high THI >65, LSMeans for MINT tended to decrease slightly again, particularly for the prompt and short-term lagged THI windows THI_0_, THI_1–3_ and THI_1–7_. In contrast, the intermediate lagged periods THI_8–14_ and THI_15–21_ maintained elevated LSMeans for MINT values across a broader THI range and showed flatter curve patterns at higher THI levels. Overall, increasing THI was associated with prolonged milking intervals, particularly under moderate and intermediate heat load conditions, whereas the behavior responses were less pronounced at very high THIs.

**Figure 7:**
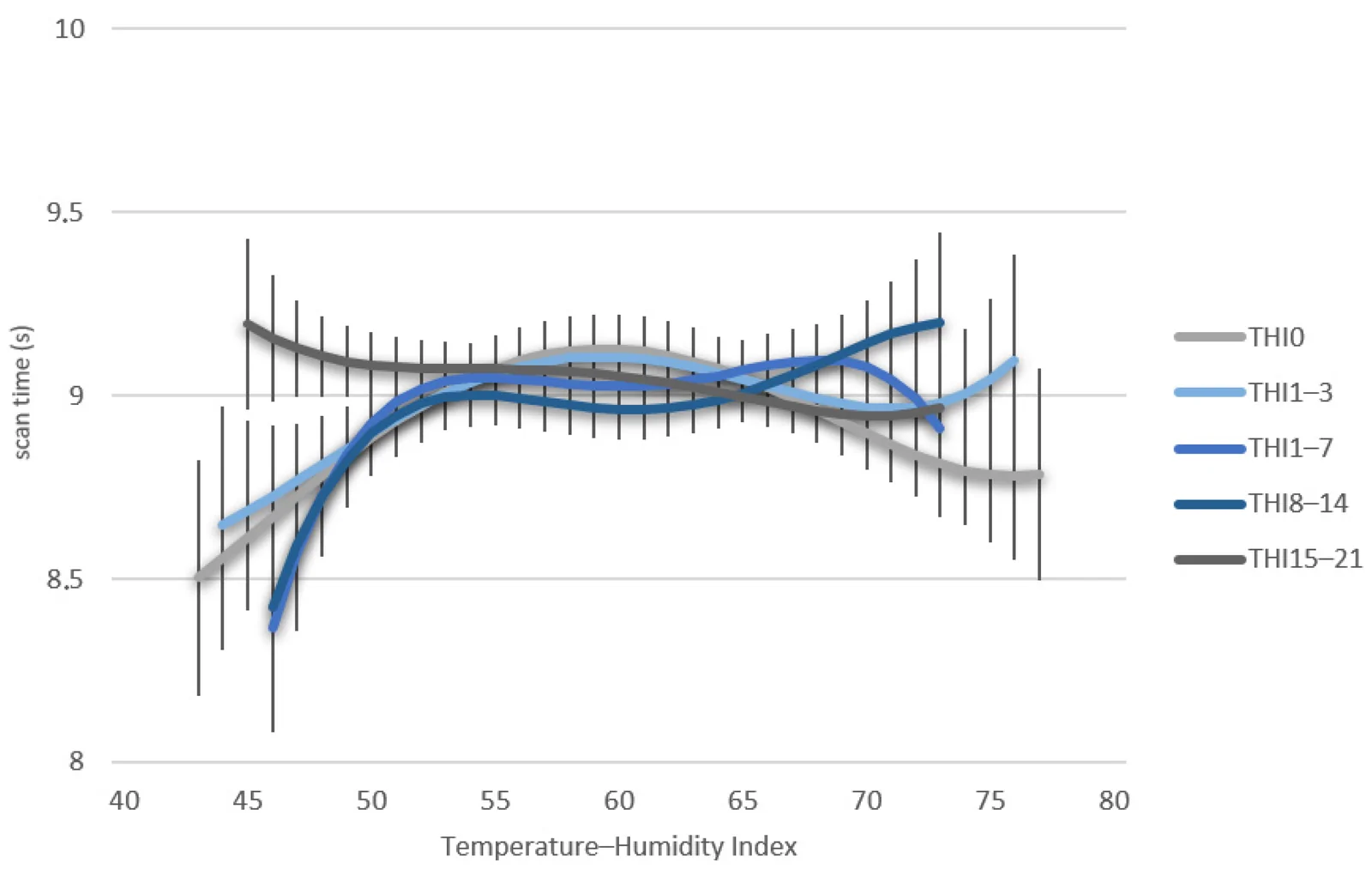
Least squares means for scan time (STIME) in seconds (s), with corresponding SEs (vertical bars) by THI from different recording periods (THI_0_, THI_1–3_, THI_1–7_, THI_8–14_, THI_15–21_) before trait recording.

Only minor variation in scan time was observed across the investigated THI range and exposure windows (Figure 7). Across all THI windows, the response curves remained relatively flat compared with the other investigated AMS traits. For the prompt and short-term lagged THI windows (THI_0_, THI_1–3_, THI_1–7_), LSMeans for STIME remained quite stable across the observed THI range, with a slight decreasing tendency at elevated THI. In contrast, the intermediate lagged periods THI_8–14_ and THI_15–21_ indicated a weak STIME increase at moderate THI, followed by a decline towards higher THI. At elevated THI, STIME differences among THI windows remained comparatively small, with similar response patterns across exposure periods. Overall, for all lagged THI windows, no clear or consistent effects of THI on STIME were observed.

**Figure 8:**
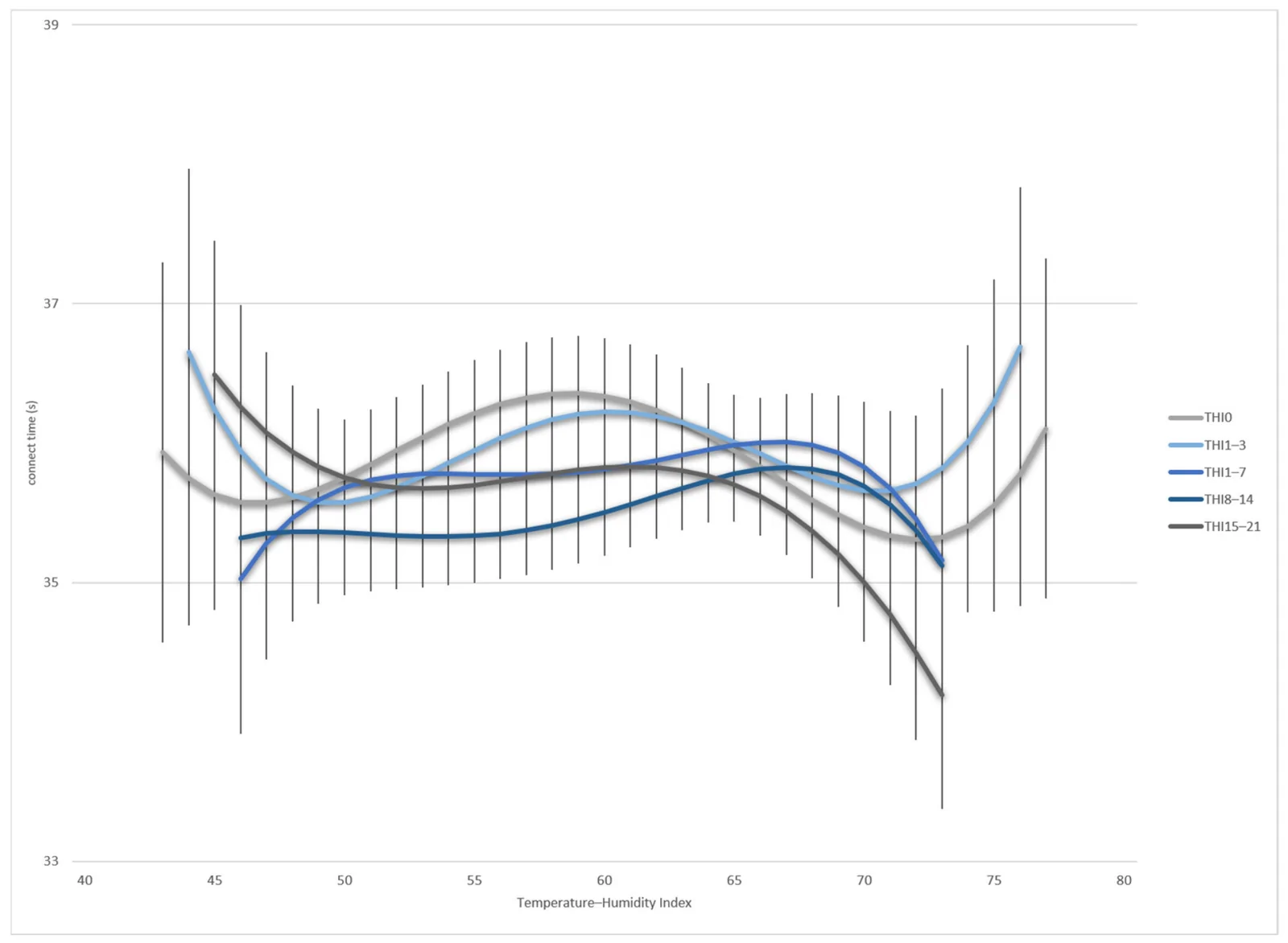
Least squares means for connect time (CTIME) in seconds (s), with corresponding SEs (vertical bars) by THI from different recording periods (THI_0_, THI_1–3_, THI_1–7_, THI_8–14_, THI_15–21_) before trait recording.

Connecting time showed a modest decline with increasing THI, with the clearest LSMeans differences observed for the short-term lagged windows (Figure 8). Compared with the AMS production traits, the magnitude of CTIME responses were comparatively small across all investigated THI windows. The most obvious CTIME decline was observed for the short-term lagged THI windows THI_1–3_ and THI_1–7_, where CTIME decreased from approximately 38 s at lower THI values to around 35 s at elevated THIs. In contrast, the prompt THI modelling approach (THI_0_) implied only minor CTIME variations across the THI range. The longer lagged periods THI_8–14_ and THI_15–21_ exhibited comparatively flat CTIME response curves with limited differentiations between low and high THI. At elevated THI, LSMeans differences between the investigated lagged periods were quite small and the trait response curves converged. Overall, the effects of THI on CTIME were comparatively weak, indicating that the most pronounced CTIME reductions were observed for short-term heat stress exposure.

**Figure 9:**
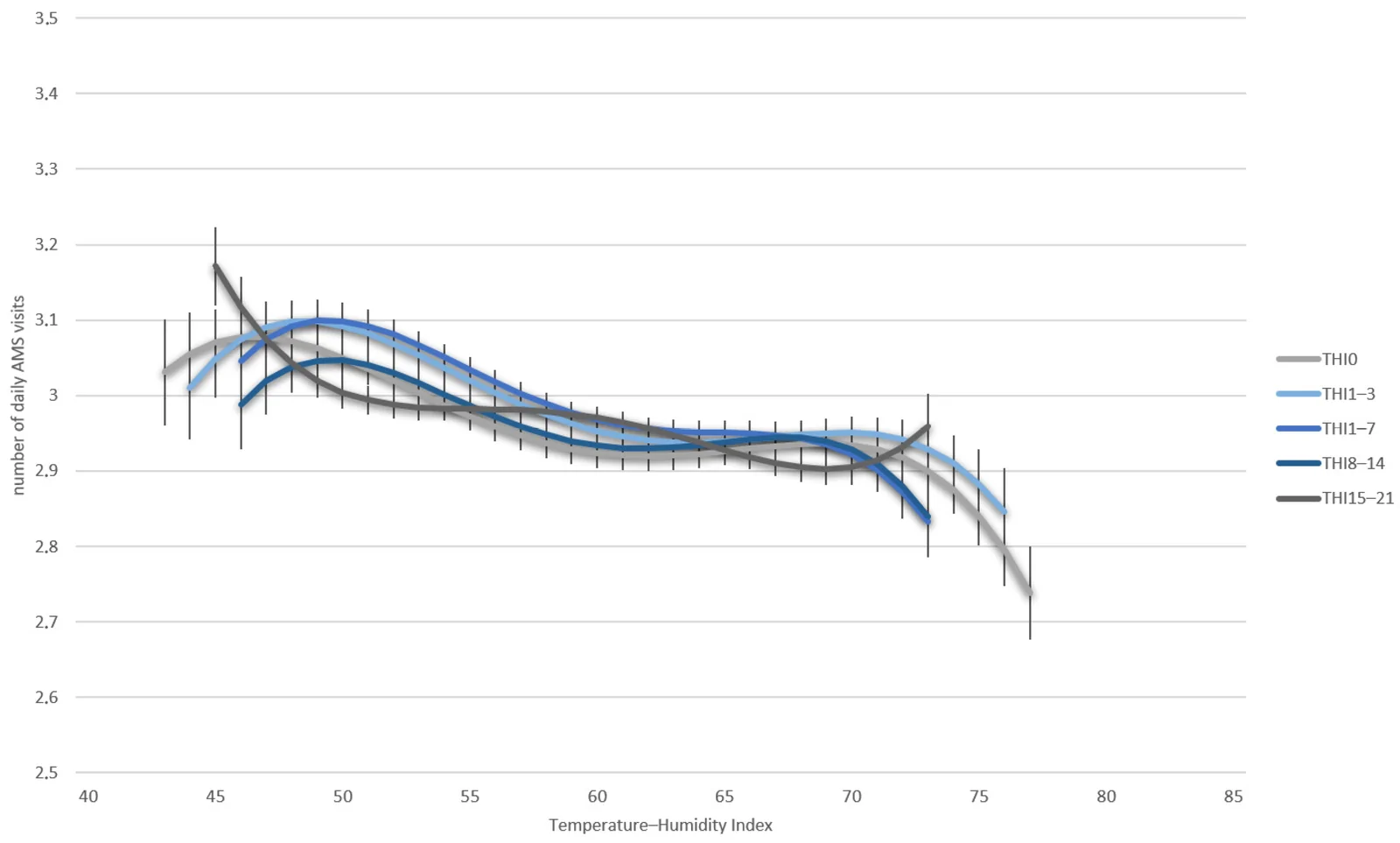
Least squares means for number of daily AMS visits (NVISITS), with corresponding SEs (vertical bars) by THI from different recording periods (THI_0_, THI_1–3_, THI_1–7_, THI_8–14_, THI_15–21_) before trait recording.

Daily AMS visit frequency decreased across the THI gradient, particularly for contemporaneous and short-term heat exposure (Figure 9). Across all investigated THI windows, NVISITS showed a consistent decline with increasing THI. Within the lower THI range from THI 40 to THI 50, cows performed around 3.0 AMS visits per day. With increasing THI, LSMeans for NVISITS declined steadily, reaching values below 2.5 visits per day at extreme THIs exceeding THI 70. The strongest declines were observed for the prompt and short-term lagged THI windows THI_0_, THI_1–3_, and THI_1–7_, which exhibited steeper response curves across the THI range. In contrast, the longer lagged periods THI_8–14_ and THI_15–21_ indicated flatter curve patterns for NVISITS, displaying minor differences between low and high THI. LSMeans for NVISITS converged at elevated THI for all of the investigated lagged THI periods, indicating consistently reduced visit frequencies under severe heat load conditions. Overall, the strongest effects of THI on NVISITS were observed for prompt and short-term heat stress exposure, whereas longer lagged periods implied weak NVISITS alterations.

### 3.3. Physiological Traits

**Figure 10:**
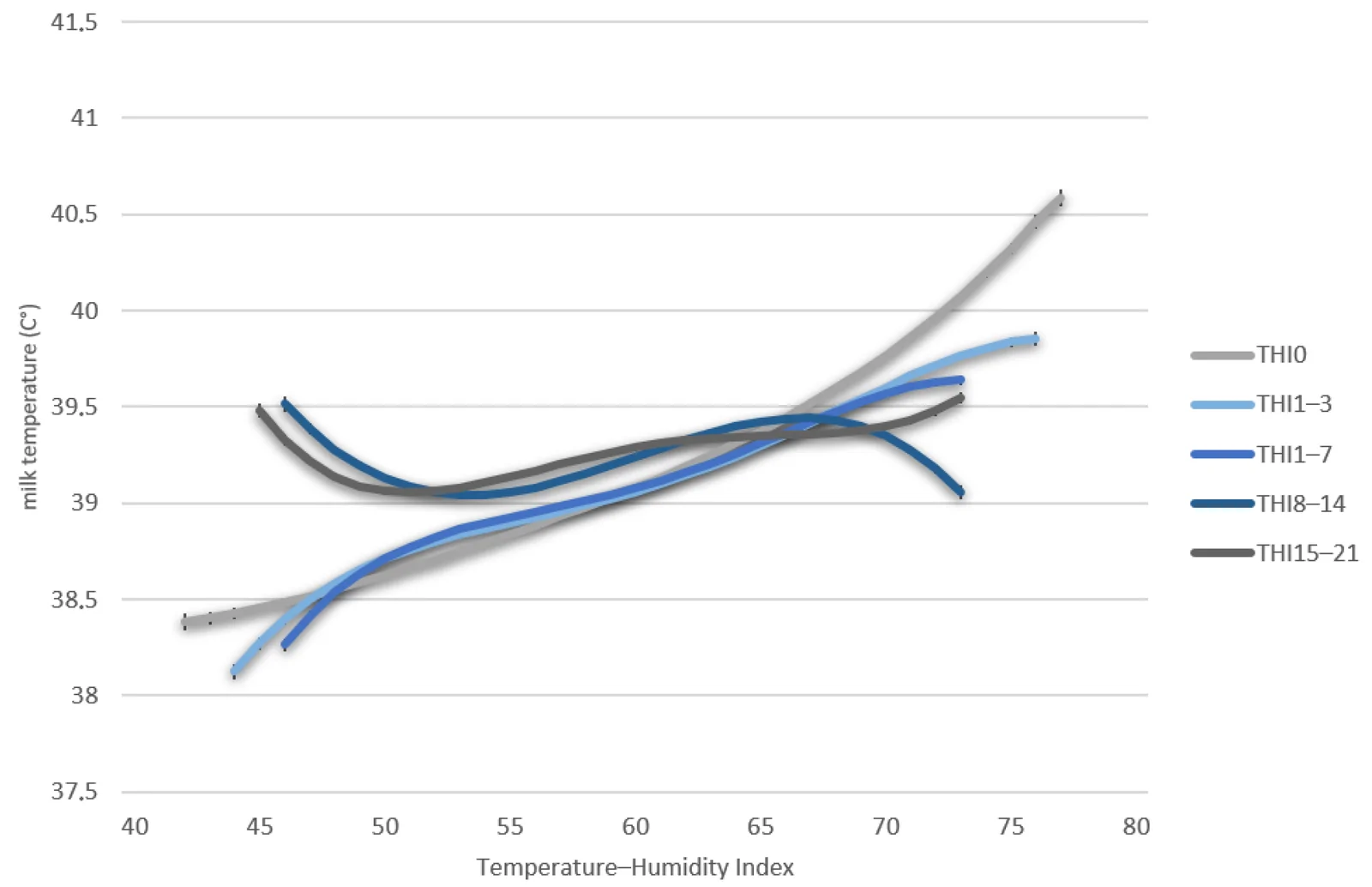
Least squares means for milk temperature (MTEMP) in degrees Celsius (°C), with corresponding SEs (vertical bars) by THI from different recording periods (THI_0_, THI_1–3_, THI_1–7_, THI_8–14_, THI_15–21_) before trait recording.

Milk temperature increased consistently with THI and showed an almost linear response in LSMeans pattern for the contemporaneous and short-term lagged exposure windows (Figure 10). For the prompt THI modelling approach (THI_0_), MTEMP increased from approximately 38.2 °C at THI 35 to more than 40.5 °C at THI exceeding 75. Similar curve patterns were observed for the short-term lagged THI windows THI_1–3_ and THI_1–7_, also indicating pronounced MTEMP increases along the THI range. In contrast, the longer lagged periods THI_8–14_ and THI_15–21_ exhibited flatter response curves and greater variability at elevated THI. The strongest increase in LSMeans for MTEMP was observed at THI 65, particularly for the prompt and short-term lagged THI windows. Overall, the strongest effects of THI on MTEMP were observed for prompt and recent heat stress exposure, whereas longer lagged periods implied flatter response patterns.

Overall, most pronounced trait response differentiations with regard to THI alterations were observed for prompt and short-term lagged THI windows (THI_0_, THI_1–3_, THI_1–7_). The longer lagged periods THI_8–14_ and THI_15–21_ mostly induced flatter response curves and less pronounced differences along the THI gradient.

## 4. Discussion

The scientific novelty of the present study is manifested in the temporally resolved integration of dense longitudinal AMS phenotypes with simultaneously recorded barn-level meteorological data. Although the unfavorable association between heat load and milk production is well established, substantially less is known about how routinely recorded AMS traits from different functional categories respond across contemporaneous and lagged THI periods under commercial farming conditions. By jointly analyzing production, behavioral, physiological, health-related, and milking-process traits across THI_0_, THI_1–3_, THI_1–7_, THI_8–14_, THI_15–21_, and THI_22–28_, the present study identified distinct temporal response patterns that would not have been apparent from official test-day records in monthly intervals. The time-window analysis is particularly relevant because heat stress responses are not instantaneous for all traits and cumulative or delayed effects may be overlooked when only prompt THI is considered. The present approach is an extension of a study combining on-farm THI measurements with longitudinal dairy cow recordings [6], demonstrating that the timing of heat-load indicators substantially influences the estimated responses of production, health, and behavioral traits.

Because feed intake, metabolic and endocrine parameters, blood flow, rumen fermentation, pathogen exposure, and sensor activity behavior were not recorded in the present study, the physiological and management-related mechanisms discussed below should be regarded as literature-supported hypotheses rather than mechanisms directly demonstrated by our data.

### 4.1. Integration of Short-Term Versus Lagged THI Effects

Across traits, the temporal structure of the THI associations suggested two broad response domains. The first comprised traits showing predominantly short-term responses, with the strongest differentiation along the THI gradient already evident for THI_0_ and remaining pronounced for THI_1–3_ and THI_1–7_. This response pattern was observed primarily for DMY, MTEMP, and NVISITS. The second domain included traits exhibiting weaker, more nonlinear, or more variable associations that became more apparent in the intermediate lag periods (THI_8–14_ and/or THI_15–21_), as observed for P%, SCS, and, to a lesser extent, MINT. This pattern is consistent with the general understanding that heat load triggers immediate thermoregulatory and behavioral responses, whereas compositional and health-related traits may reflect downstream metabolic and immunological processes developing over longer time periods [8,20].

The longest lag period (THI_22–28_) was also evaluated but is not presented in the LSMeans figures because it did not provide additional explanatory value beyond the shorter THI windows. The corresponding models generally showed flatter and less distinct response patterns across the investigated traits, suggesting that heat stress occurring more than three weeks before the recording day had little direct influence on the evaluated AMS-derived traits. This observation is biologically plausible, as most within-lactation heat stress effects on milk yield and milking behavior emerge rapidly and decline within days to approximately two weeks, consistent with previous transfer-function and time-lag studies demonstrating concentrated short-lag responses for milk yield [21] and unfavorable effects of THI on milking frequency within a few days in AMS [22].

Nevertheless, this finding does not imply that heat stress lacks longer-term consequences. Carry-over effects are well documented for heat exposure during sensitive physiological periods such as late gestation and the dry period, where heat stress may influence mammary development, immune function, and subsequent lactation performance [23]. Rather, the present results indicate that a within-lactation THI_22–28_ window appears too distant to explain variation in the AMS-derived traits recorded on the focal day.

### 4.2. Production and Composition Responses

Daily milk yield declined consistently with increasing THI, with the clearest differentiation among THI in THI_0_ and short-lagged periods (THI_1–3_, T_1–7_THI_1–7_) but displaying minor effects in THI_15–21_.

The decline in DMY with increasing THI is consistent with mechanisms reported in previous experimental studies, including reduced dry matter intake, increased maintenance requirements for thermoregulation, and altered endocrine–metabolic nutrient partitioning under hyperthermia [7,20]. Previous studies additionally described panting, sweating, and peripheral vasodilation, together with the associated energy and water requirements, as possible contributors to heat-related production losses [8,24]. Consequently, cows kept in temperate production systems may exhibit measurable production losses at THI values below the commonly cited threshold [25].

Milk components showed nonlinear and lag-dependent responses. The observed tendency for P% to decline at higher THI may be consistent with heat-related changes in nutrient availability and metabolic partitioning reported in previous studies [20]. For F%, the inverted U-shaped pattern (higher values at intermediate THI, lower at high THI) could reflect several interacting mechanisms. Reduced milk yield at moderate heat stress may initially increase fat concentration through a concentration effect, whereas more severe heat stress is accompanied by reduced dry matter intake, altered rumen fermentation, and decreased acetate availability, thereby limiting de novo fatty acid synthesis in the mammary gland [20,26]. Feed intake, rumen fermentation, and milk-component yields were not measured in the present study. Respective heat stress effects in a causal context are assumed, but direct measurements of these traits are imperative for validations in this regard. Field studies further indicated that milk-fat responses may be less stable without displaying consistent THI thresholds [25,27].

### 4.3. Behavioral Mediation and Milking-Process Traits in AMS

The consistent reduction in NVISITS with increasing THI suggests that behavioral changes in voluntary cow traffic may represent one pathway through which heat load translates into production efficiency in AMS settings. Studies in pasture-based and temperate-climate AMS systems have similarly reported reductions in milking frequency and performance associated with increasing THI, often with short time lags of one to two days [25]. More broadly, sensor-based studies have shown that behavioral responses to heat stress, including reduced feeding and walking activity as well as increased standing time, become detectable already at relatively moderate THI levels in temperate production systems [28]. The increase in MINT with increasing THI, peaking at intermediate THI levels and weakening at the highest THI values, likely reflects a combination of reduced motivation to visit the robot, altered daily activity patterns, and management-related responses such as increased fetching or modified cow flow during hot periods.

Importantly, the observed reductions in NVISITS together with prolonged MINT provide a plausible behavioral link between heat load and reduced milk production, as fewer voluntary visits and longer milking intervals are expected to decrease effective milking frequency, which is closely associated with milk yield in AMS herds [29,30]. However, because the present study was observational, the direction of these associations cannot be determined unequivocally. Reduced robot attendance may contribute to lower milk yield, whereas declining milk yield may similarly influence a cow’s motivation or eligibility to visit the AMS. Consequently, the observed relationships should be interpreted as associations rather than evidence of causal mediation.

In contrast to the clear behavioral responses observed for NVISITS and MINT, the associations of STIME and CTIME with THI were comparatively weak and less consistent across lagged THI windows. This suggests that heat stress in AMS environments primarily affects cow behavior and physiology rather than substantially disrupting the technical milking process itself. Although CTIME declined slightly with increasing THI, STIME remained comparatively stable, indicating that teat detection and attachment procedures were largely robust under varying climatic conditions.

The modest CTIME response may nevertheless reflect subtle changes in cow–robot interaction during heat stress. Connecting time is influenced not only by AMS performance, but also by cow posture, movement behavior, udder conformation, and willingness to stand calmly during attachment [7,8]. Previous AMS studies further demonstrated that milking-process traits are generally characterized by greater between-cow variability and lower environmental sensitivity than production or behavioral traits [30]. Consequently, cow-specific anatomical and behavioral factors may exert stronger influences on STIME and CTIME than short-term climatic variation alone [31].

Overall, the findings suggest that the primary heat-stress pathway in AMS systems operates through altered cow behavior and voluntary robot attendance, whereas the robotic milking process itself remains comparatively resilient under moderate climatic stress conditions.

### 4.4. MTEMP and SCS: Immediate Heat Strain Versus Udder-Health Dynamics

Milk temperature increased sharply and near-linearly with THI, with the clearest signal in THI_0_ and short-lagged THI windows. This is consistent with the concept that animal-based temperature proxies react promptly to heat load and can provide a more immediate reflection of cow heat strain than performance outcomes alone. Indeed, comprehensive reviews of heat-stress indicators highlight body temperature as one of the most direct non-invasive measures of individual heat load, whereas yield traits are often interpreted as downstream consequences [32]. In AMS practice, MTEMP could therefore complement THI-based alerts, particularly where barn microclimate differs from regional weather-station data, and may help distinguish current heat strain from downstream production changes. Altınsoy et al. observed clear seasonal increases in AMS-derived MTEMP in warmer months and described MTEMP as a proxy phenotype for short-term thermoregulatory/metabolic variation (not a direct core-temperature measure), based on a single-herd dataset [33].

Somatic cell score showed an overall tendency to increase with increasing THI, but with substantially greater variability across THI windows and at higher THI. Heterogeneity in the SCS responses is consistent with the multifactorial nature of udder health during heat load. Potential drivers include immunomodulation under heat stress, increased pathogen pressure in warm and humid conditions, changes in lying/standing behavior that affect teat-end contamination risk, and mechanical/physiological effects of prolonged milking intervals. Published time-lagged analyses suggest that SCS responses occur with longer lags than milk yield; in large datasets, the lag for SCS has been reported to extend beyond the peak milk-yield lag, supporting the plausibility of more delayed or distributed effects [34].

### 4.5. Implications for AMS Management and Future Research

From an AMS management perspective, the joint pattern of decreasing NVISITS and increasing MINT highlights cow traffic as a critical operational vulnerability during heat load. Practical mitigation may therefore benefit from focusing cooling and comfort resources not only in resting areas but also along cow-traffic routes and especially near the robot/waiting zone, aiming to preserve voluntary attendance and avoid extended intervals. The strong MTEMP response suggests that integrating MTEMP into on-farm monitoring could support the earlier detection of heat-load periods and more timely interventions, consistent with calls to implement animal-based indicators alongside climate indices [24,32]. The pronounced phenotypic MTEMP variability was confirmed in recent genetic studies, indicating a strong additive-genetic component for MTEMP and substantial MTEMP breeding value alterations [35]. Hence, it is suggested to use MTEMP as a predominant response trait when developing heat tolerance breeding indices.

Beyond these practical implications, the DAIRY CHAOS procedure applies two cow-specific anomaly-detection algorithms to identify unusual daily production and behavioral patterns associated with periods of heat load [36]. Individual susceptibility to heat stress can also be investigated using generalized additive models with mixed effects. Benni et al. [37] combined data from AMS and related monitoring devices with barn-climate measurements and used cow-specific random effects for the intercept and THI response to distinguish cows with significant, moderate, or poor susceptibility to heat stress. Such approaches account for heterogeneity among cows and may support the identification of particularly vulnerable animals and the implementation of targeted cooling and management strategies. In contrast, the present mixed-model approach was designed to describe and compare population-level THI response patterns across multiple AMS-derived traits and predefined exposure windows. These approaches should therefore be considered complementary and could be combined in future studies to develop individual-cow heat-stress monitoring systems.

From a management perspective, ongoing research priorities should focus on (i) multi-herd validation, particularly for SCS; (ii) linking THI windows to additional animal-based sensors (core/reticular temperature, rumination and feeding time) to improve mechanistic attribution; and (iii) applying distributed-lag nonlinear modeling approaches to quantify response shapes and uncertainty across lagged THI windows. In temperate regions, where behavioral adaptations may begin at relatively low THI [38], refined farm-specific thresholding and integrated cow-side monitoring appear especially warranted.

### 4.6. Study Limitations

The data originated from two commercial dairy farms located in North Rhine-Westphalia, and one commercial dairy farm located in Hesse, Germany. Hence, not all German federal states are represented with AMS and climate data in the present study, and all herds consisted of kept German Holstein cows managed in AMS with voluntary cow traffic. The observed associations between THI and cow responses may therefore not be directly transferable to other climatic regions, breeds, housing systems, or management conditions. In particular, differences in barn design, cooling strategies, feeding management, cow traffic, and regional climatic conditions may modify the magnitude and temporal pattern of heat-stress responses. Accordingly, comparisons with studies conducted in pasture-based systems, warmer climatic regions, or herds with different cow-traffic and cooling strategies should be interpreted cautiously [1,22,25,26,38]. Similarities in the direction of trait responses do not necessarily imply comparable response magnitudes or THI thresholds across climatic regions and production systems. Nevertheless, the present study is based on a comprehensive dataset, and Lely milking robot systems, as used in the present study, are representative for most of the German AMS herds.

Second, the observational nature of this study does not allow inferring causal relationships between heat load and the investigated AMS-derived trait responses. Although the statistical models accounted for relevant systematic and cow-specific effects, unmeasured management or environmental factors may have contributed to the observed associations. Consequently, the identified THI response patterns should be interpreted as associations under the investigated commercial farming conditions rather than as universally applicable biological thresholds. Future studies including a larger number of herds across different climatic regions, breeds, and management systems are required to assess the generalizability of these findings.

## 5. Conclusions

The present study demonstrates that combining dense longitudinal AMS data with continuously recorded on-farm meteorological measurements provides detailed insights into both immediate and delayed heat stress responses in dairy cows. Increasing THI was associated with reduced daily milk yield, altered milk composition, lower AMS visit frequency, prolonged milking intervals, and increased milk temperature. The strongest and most consistent trait responses were generally observed due to prompt and short-term lagged THI effects, whereas longer lagged periods implied weaker associations. In particular, milk temperature proved to be highly responsive to increasing THI, whereas production and behavioral traits showed distinct trait-specific response patterns across the investigated lagged periods.

Although the present modelling approach was not designed to estimate discrete biological THI thresholds, the response curves identified practically relevant THI ranges. Pronounced reductions in DMY were already apparent between THI 45 and 55, whereas MINT increased markedly within a similar range and reached its highest levels at approximately THI 55–65. Changes in milk composition were nonlinear, with F% and P% reaching maximum values around THI 50–55 before declining at higher THI. MTEMP showed a continuous positive response to increasing THI, with particularly pronounced increases at THI values above approximately 65. SCS responses were more variable, particularly at high THI, and therefore did not support the identification of a distinct threshold.

The results highlight the potential of integrating high-frequency AMS-derived phenotypes with farm-specific climatic data for precision heat stress monitoring under practical dairy farming conditions and for ongoing breeding approaches on improved heat tolerance. Compared with conventional low-frequency recording systems and weather-station-based approaches, the use of dense on-farm data allows a more detailed characterization of temporal heat stress dynamics and associated cow responses. These findings may support the development of improved management strategies and data-driven monitoring tools to mitigate the heat stress impacts on dairy cow productivity, welfare, and health under increasing climate variability.

## Figures and Tables

**Table 1:** Descriptive statistics of AMS-derived production, physiological, behavioral and milking-process traits included in the analyses. SD = standard deviation.

Trait	Abbreviation	Mean	SD	Min	Max
Daily milk yield (kg)	DMY	34.72	11.04	3.00	70.00
Milk fat percentage (%)	F%	3.92	0.83	1.50	7.00
Milk protein percentage (%)	P%	3.38	0.28	2.40	4.30
Somatic cell score	SCS	2.78	2.31	0	8.42
Milk temperature (°C)	MTEMP	39.30	0.66	36.00	42.00
Number of AMS visits per day	NVISITS	3.01	0.83	1.00	5.00
Milking interval (h)	MINT	8.04	2.42	4.66	18.00
Scan time (s)	STIME	9.56	4.72	1.00	60.00
Connecting time (s)	CTIME	38.45	21.44	10.00	120.00

## Data Availability

Data supporting this study are available upon request of the authors.

## Supplementary Materials

The following supporting information can be downloaded at: https://www.mdpi.com/article/10.3390/ani16172671/s1, Supplementary File S1: Representative detailed SAS outputs for milk protein percentage (P%), milking interval (MINT), and milk temperature (MTEMP) for the THI_1–3_ exposure window.

## Abbreviations

The following abbreviations are used in this manuscript:

AMS	Automatic Milking System
CTIME	Connecting Time
DMY	Daily Milk Yield
F%	Milk Fat Percentage
MINT	Milking Interval
MTEMP	Milk Temperature
NVISITS	Number of Daily AMS Visits
P%	Milk Protein Percentage
RH	Relative Humidity
SCS	Somatic Cell Score
SD	Standard Deviation
SE	Standard Error
STIME	Scan Time
Temp	Ambient Temperature
THI	Temperature–Humidity Index
THI_0_	Temperature–Humidity Index of the Recording Day
THI_1–3_	Average Temperature–Humidity Index 1–3 Days before Recording
THI_1–7_	Average Temperature–Humidity Index 1–7 Days before Recording
THI_8–14_	Average Temperature–Humidity Index 8–14 Days before Recording
THI_15–21_	Average Temperature–Humidity Index 15–21 Days before Recording
THI_22–28_	Average Temperature–Humidity Index 22–28 Days before Recording
