# Supplementary material for: Combining Dense Longitudinal Records from Robotic Milking with Dense On-Farm Meteorological Data to Assess Heat Stress Effects in Dairy Cows
Source: Animals (Basel). 2026 Aug 25;16(17):2671. doi: 10.3390/ani16172671 (PMC13564302; doi:10.3390/ani16172671)
Supplement: Supplementary file 1 [file animals-16-02671-s001.zip › animals-4456896-supplementary.pdf]

Supplementary File S1
Representative detailed SAS outputs for selected statistical analyses

Supplementary material for:
Combining dense longitudinal records from robotic milking with dense on-farm meteorological data to assess heat stress effects in dairy cows

Results for milking interval (MINT) from the period 1-3 days before trait recording

The Mixed Procedure

Model Information

Data Set	WORK.AMS
Dependent Variable	interval
Covariance Structure	Variance Components
Subject Effect	Cow
Estimation Method	REML
Residual Variance Method	Profile
Fixed Effects SE Method	Model-Based
Degrees of Freedom Method	Satterthwaite

Class Level Information

Class	Levels	Values
Lac	5	1 2 3 4 5
Herd	3	A B C
HYS	12	A20223 A20232 B20232 B20233 B20242 B20243 C20232 C20233 C20242 C20243 C20252 C20253
Robot	8	63 64 65 66 67 69 86 87
Cow	789	...
dummy	1	1
Groupsize	3	<40 >=55 40-55
Daytime	3	Nacht Vormittag Nachmittag

Dimensions

Covariance Parameters	5
Columns in X	42
Columns in Z Per Subject	4
Subjects	789
Max Obs Per Subject	1776

Number of Observations

Number of Observations Read	381236
Number of Observations Used	377921
Number of Observations Not Used	3315

Iteration History

Iteration	Evaluations	-2 Res Log Like	Criterion
0	1	1691761.3206079	
1	2	1607650.6235442	0.00272194
2	1	1605993.4770094	0.00179842
3	1	1604908.9757775	0.00110401
4	1	1604247.5394684	0.00063801
5	1	1603868.3035750	0.00033280
6	1	1603673.6385069	0.00014137
7	1	1603593.3597677	0.00004034
8	1	1603571.6612505	0.00000505
9	1	1603569.1643158	0.00000011
10	1	1603569.1143277	0.00000000

Convergence criteria met.

Covariance Parameter Estimates

Cov Parm	Subject	Estimate
lg_thi1_3_1	Cow	5.6553
lg_thi1_3_2	Cow	8.4306
lg_thi1_3_3	Cow	2.9281
lg_thi1_3_4	Cow	3.0341
Residual		3.9579

Fit Statistics

-2 Res Log Likelihood	1603569
AIC (smaller is better)	1603579
AICC (smaller is better)	1603579
BIC (smaller is better)	1603602

Type 1 Tests of Fixed Effects

Effect	Num DF	Den DF	F Value	Pr > F
dummy	1	1615	66171.2	<.0001
lg_thi1_3_1	1	1170	19.81	<.0001
lg_thi1_3_2	1	929	11.08	0.0009
lg_thi1_3_3	1	886	0.06	0.8100
lg_thi1_3_4	1	774	0.16	0.6893
Lac	4	28E4	860.39	<.0001
lg_dim_1	1	35E4	3254.04	<.0001
lg_dim_2	1	37E4	3614.14	<.0001
lg_dim_3	1	38E4	729.31	<.0001
lg_dim_4	1	38E4	1756.47	<.0001
HYS(Herd)	11	26E4	207.91	<.0001
Robot(Herd)	7	25E4	457.50	<.0001
Groupsize	2	38E4	199.21	<.0001
Daytime	2	37E4	7855.85	<.0001

Type 3 Tests of Fixed Effects

Effect	Num DF	Den DF	F Value	Pr > F
dummy	0	.	.	.
lg_thi1_3_1	1	776	43.77	<.0001
lg_thi1_3_2	1	831	14.36	0.0002
lg_thi1_3_3	1	758	1.44	0.2300
lg_thi1_3_4	1	787	0.63	0.4270
Lac	4	28E4	791.50	<.0001
lg_dim_1	1	35E4	3926.36	<.0001
lg_dim_2	1	37E4	3588.55	<.0001
lg_dim_3	1	38E4	552.49	<.0001
lg_dim_4	1	38E4	1492.82	<.0001
HYS(Herd)	9	36E4	172.02	<.0001
Robot(Herd)	7	25E4	305.38	<.0001
Groupsize	2	38E4	209.22	<.0001
Daytime	2	37E4	7855.85	<.0001

Least Squares Means

Effect	dummy	lg_thi1_3_1	lg_thi1_3_2	lg_thi1_3_3	lg_thi1_3_4	lg_dim_1	lg_dim_2	lg_dim_3	lg_dim_4	Estimate	Standard Error	DF	t Value	Pr > |t|
dummy	1	-1.00	1.00	-1.00	1.00	-0.02	-0.03	-0.01	-0.00	7.2189	0.1868	3028	38.64	<.0001
dummy	1	-0.94	0.82	-0.65	0.46	-0.02	-0.03	-0.01	-0.00	7.3293	0.1442	2568	50.82	<.0001
dummy	1	-0.88	0.65	-0.36	0.07	-0.02	-0.03	-0.01	-0.00	7.4386	0.1138	2038	65.35	<.0001
dummy	1	-0.81	0.49	-0.12	-0.19	-0.02	-0.03	-0.01	-0.00	7.5459	0.09346	1684	80.74	<.0001
dummy	1	-0.75	0.34	0.07	-0.35	-0.02	-0.03	-0.01	-0.00	7.6501	0.08052	1507	95.01	<.0001
dummy	1	-0.69	0.21	0.22	-0.42	-0.02	-0.03	-0.01	-0.00	7.7506	0.07232	1448	107.17	<.0001
dummy	1	-0.63	0.09	0.33	-0.42	-0.02	-0.03	-0.01	-0.00	7.8466	0.06672	1484	117.60	<.0001
dummy	1	-0.56	-0.03	0.40	-0.37	-0.02	-0.03	-0.01	-0.00	7.9376	0.06248	1621	127.05	<.0001
dummy	1	-0.50	-0.13	0.44	-0.29	-0.02	-0.03	-0.01	-0.00	8.0229	0.05911	1856	135.72	<.0001
dummy	1	-0.44	-0.21	0.45	-0.18	-0.02	-0.03	-0.01	-0.00	8.1022	0.05663	2113	143.07	<.0001
dummy	1	-0.38	-0.29	0.43	-0.07	-0.02	-0.03	-0.01	-0.00	8.1750	0.05517	2192	148.19	<.0001
dummy	1	-0.31	-0.35	0.39	0.05	-0.02	-0.03	-0.01	-0.00	8.2412	0.05477	1968	150.48	<.0001
dummy	1	-0.25	-0.41	0.34	0.16	-0.02	-0.03	-0.01	-0.00	8.3006	0.05524	1620	150.26	<.0001
dummy	1	-0.19	-0.45	0.26	0.25	-0.02	-0.03	-0.01	-0.00	8.3529	0.05623	1336	148.55	<.0001
dummy	1	-0.13	-0.48	0.18	0.32	-0.02	-0.03	-0.01	-0.00	8.3984	0.05729	1155	146.60	<.0001
dummy	1	-0.06	-0.49	0.09	0.36	-0.02	-0.03	-0.01	-0.00	8.4370	0.05803	1055	145.40	<.0001
dummy	1	0.00	-0.50	0.00	0.38	-0.02	-0.03	-0.01	-0.00	8.4688	0.05816	1018	145.60	<.0001
dummy	1	0.06	-0.49	-0.09	0.36	-0.02	-0.03	-0.01	-0.00	8.4943	0.05760	1032	147.48	<.0001
dummy	1	0.13	-0.48	-0.18	0.32	-0.02	-0.03	-0.01	-0.00	8.5136	0.05638	1102	151.00	<.0001
dummy	1	0.19	-0.45	-0.26	0.25	-0.02	-0.03	-0.01	-0.00	8.5274	0.05476	1242	155.71	<.0001
dummy	1	0.25	-0.41	-0.34	0.16	-0.02	-0.03	-0.01	-0.00	8.5359	0.05314	1466	160.62	<.0001

dummy	1	0.31	-0.35	-0.39	0.05	-0.02	-0.03	-0.01	-0.00	8.5400	0.05203	1737	164.14	<.0001
dummy	1	0.38	-0.29	-0.43	-0.07	-0.02	-0.03	-0.01	-0.00	8.5403	0.05191	1885	164.51	<.0001
dummy	1	0.44	-0.21	-0.45	-0.18	-0.02	-0.03	-0.01	-0.00	8.5375	0.05312	1770	160.73	<.0001
dummy	1	0.50	-0.13	-0.44	-0.29	-0.02	-0.03	-0.01	-0.00	8.5326	0.05567	1540	153.26	<.0001
dummy	1	0.56	-0.03	-0.40	-0.37	-0.02	-0.03	-0.01	-0.00	8.5265	0.05941	1369	143.53	<.0001
dummy	1	0.63	0.09	-0.33	-0.42	-0.02	-0.03	-0.01	-0.00	8.5202	0.06415	1291	132.82	<.0001
dummy	1	0.69	0.21	-0.22	-0.42	-0.02	-0.03	-0.01	-0.00	8.5149	0.07007	1288	121.52	<.0001
dummy	1	0.75	0.34	-0.07	-0.35	-0.02	-0.03	-0.01	-0.00	8.5119	0.07797	1334	109.16	<.0001
dummy	1	0.81	0.49	0.12	-0.19	-0.02	-0.03	-0.01	-0.00	8.5124	0.08953	1437	95.08	<.0001
dummy	1	0.88	0.65	0.36	0.07	-0.02	-0.03	-0.01	-0.00	8.5178	0.1071	1666	79.51	<.0001
dummy	1	0.94	0.82	0.65	0.46	-0.02	-0.03	-0.01	-0.00	8.5296	0.1334	2070	63.93	<.0001
dummy	1	1.00	1.00	1.00	1.00	-0.02	-0.03	-0.01	-0.00	8.5495	0.1708	2494	50.06	<.0001

Results for milk temperature (MTEMP) from the period 1-3 days before trait recording

The Mixed Procedure

Model Information

Data Set	WORK.AMS
Dependent Variable	milktemp
Covariance Structure	Variance Components
Subject Effect	Cow
Estimation Method	REML
Residual Variance Method	Profile
Fixed Effects SE Method	Model-Based
Degrees of Freedom Method	Satterthwaite

Class Level Information

Class	Levels	Values
Lac	5	1 2 3 4 5
Herd	3	A B C
HYS	12	A20223 A20232 B20232 B20233 B20242 B20243 C20232 C20233 C20242 C20243 C20252 C20253
Robot	8	63 64 65 66 67 69 86 87
Cow	790	...
dummy	1	1
Groupsize	3	<40 >=55 40-55
Daytime	3	Nacht Vormittag Nachmittag

Dimensions

Covariance Parameters	5
Columns in X	38
Columns in Z Per Subject	4
Subjects	790
Max Obs Per Subject	1776

Number of Observations

Number of Observations Read	386587
Number of Observations Used	345801
Number of Observations Not Used	40786

Iteration History

Iteration	Evaluations	-2 Res Log Like	Criterion
0	1	523694.85471424	
1	3	468059.02242519	0.00845907
2	1	467119.55855563	0.00529310
3	1	466532.37537234	0.00309706
4	1	466189.84597783	0.00165902
5	1	466008.41321741	0.00074444
6	1	465928.99488691	0.00023658
7	1	465904.92832161	0.00003715
8	1	465901.43848458	0.00000129
9	1	465901.32625742	0.00000000

Convergence criteria met.

Covariance Parameter Estimates

Cov Parm	Subject	Estimate
lg_thi1_3_1	Cow	0.1901
lg_thi1_3_2	Cow	0.3210
lg_thi1_3_3	Cow	0.09612
lg_thi1_3_4	Cow	0.09525
Residual		0.2198

Fit Statistics

-2 Res Log Likelihood	465901.3
AIC (smaller is better)	465911.3
AICC (smaller is better)	465911.3
BIC (smaller is better)	465934.7

Type 1 Tests of Fixed Effects

Effect	Num DF	Den DF	F Value	Pr > F
dummy	1	1293	3.176E7	<.0001
lg_thi1_3_1	1	1131	1484.12	<.0001
lg_thi1_3_2	1	729	25.89	<.0001
lg_thi1_3_3	1	828	1.20	0.2731
lg_thi1_3_4	1	693	69.72	<.0001
Lac	4	24E4	1073.57	<.0001
lg_dim_1	1	29E4	2715.24	<.0001
lg_dim_2	1	34E4	665.36	<.0001
lg_dim_3	1	34E4	11.92	0.0006
lg_dim_4	1	34E4	17.16	<.0001
HYS(Herd)	9	29E4	258.83	<.0001
Robot(Herd)	6	34E4	6669.19	<.0001
Groupsize	2	34E4	169.26	<.0001
Daytime	2	34E4	14872.0	<.0001

Type 3 Tests of Fixed Effects

Effect	Num DF	Den DF	F Value	Pr > F
dummy	0	.	.	.
lg_thi1_3_1	1	670	1829.22	<.0001
lg_thi1_3_2	1	689	0.04	0.8476
lg_thi1_3_3	1	664	47.73	<.0001
lg_thi1_3_4	1	707	72.52	<.0001
Lac	4	24E4	463.24	<.0001
lg_dim_1	1	29E4	3622.80	<.0001
lg_dim_2	1	34E4	872.68	<.0001
lg_dim_3	1	34E4	36.66	<.0001
lg_dim_4	1	34E4	2.15	0.1424
HYS(Herd)	8	33E4	199.80	<.0001
Robot(Herd)	6	34E4	6631.10	<.0001
Groupsize	2	34E4	164.00	<.0001
Daytime	2	34E4	14872.0	<.0001

Least Squares Means

Effect	dummy	lg_thi1_3_1	lg_thi1_3_2	lg_thi1_3_3	lg_thi1_3_4	lg_dim_1	lg_dim_2	lg_dim_3	lg_dim_4	Estimate	Standard Error	DF	t Value	Pr > |t|
dummy	1	-1.00	1.00	-1.00	1.00	-0.01	-0.02	-0.01	-0.00	38.1268	0.04090	2850	932.20	<.0001
dummy	1	-0.94	0.82	-0.65	0.46	-0.01	-0.02	-0.01	-0.00	38.2728	0.03148	2447	1215.84	<.0001
dummy	1	-0.88	0.65	-0.36	0.07	-0.01	-0.02	-0.01	-0.00	38.3953	0.02461	1975	1560.22	<.0001
dummy	1	-0.81	0.49	-0.12	-0.19	-0.01	-0.02	-0.01	-0.00	38.4975	0.01990	1630	1934.85	<.0001
dummy	1	-0.75	0.34	0.07	-0.35	-0.01	-0.02	-0.01	-0.00	38.5824	0.01686	1433	2287.85	<.0001
dummy	1	-0.69	0.21	0.22	-0.42	-0.01	-0.02	-0.01	-0.00	38.6526	0.01498	1346	2580.09	<.0001
dummy	1	-0.63	0.09	0.33	-0.42	-0.01	-0.02	-0.01	-0.00	38.7109	0.01378	1363	2809.16	<.0001
dummy	1	-0.56	-0.03	0.40	-0.37	-0.01	-0.02	-0.01	-0.00	38.7595	0.01295	1493	2993.02	<.0001
dummy	1	-0.50	-0.13	0.44	-0.29	-0.01	-0.02	-0.01	-0.00	38.8006	0.01234	1729	3143.71	<.0001
dummy	1	-0.44	-0.21	0.45	-0.18	-0.01	-0.02	-0.01	-0.00	38.8362	0.01192	1966	3258.37	<.0001
dummy	1	-0.38	-0.29	0.43	-0.07	-0.01	-0.02	-0.01	-0.00	38.8681	0.01169	1962	3326.20	<.0001
dummy	1	-0.31	-0.35	0.39	0.05	-0.01	-0.02	-0.01	-0.00	38.8979	0.01164	1660	3341.65	<.0001
dummy	1	-0.25	-0.41	0.34	0.16	-0.01	-0.02	-0.01	-0.00	38.9271	0.01175	1307	3313.22	<.0001
dummy	1	-0.19	-0.45	0.26	0.25	-0.01	-0.02	-0.01	-0.00	38.9568	0.01195	1053	3261.24	<.0001
dummy	1	-0.13	-0.48	0.18	0.32	-0.01	-0.02	-0.01	-0.00	38.9881	0.01215	900	3209.14	<.0001
dummy	1	-0.06	-0.49	0.09	0.36	-0.01	-0.02	-0.01	-0.00	39.0218	0.01229	819	3176.20	<.0001
dummy	1	0.00	-0.50	0.00	0.38	-0.01	-0.02	-0.01	-0.00	39.0586	0.01230	787	3175.18	<.0001
dummy	1	0.06	-0.49	-0.09	0.36	-0.01	-0.02	-0.01	-0.00	39.0990	0.01217	794	3212.63	<.0001
dummy	1	0.13	-0.48	-0.18	0.32	-0.01	-0.02	-0.01	-0.00	39.1431	0.01190	843	3289.22	<.0001
dummy	1	0.19	-0.45	-0.26	0.25	-0.01	-0.02	-0.01	-0.00	39.1911	0.01153	945	3398.25	<.0001
dummy	1	0.25	-0.41	-0.34	0.16	-0.01	-0.02	-0.01	-0.00	39.2429	0.01114	1120	3522.37	<.0001
dummy	1	0.31	-0.35	-0.39	0.05	-0.01	-0.02	-0.01	-0.00	39.2982	0.01082	1361	3630.73	<.0001

dummy	1	0.38	-0.29	-0.43	-0.07	-0.01	-0.02	-0.01	-0.00	39.3563	0.01068	1543	3683.34	<.0001
dummy	1	0.44	-0.21	-0.45	-0.18	-0.01	-0.02	-0.01	-0.00	39.4168	0.01081	1480	3647.94	<.0001
dummy	1	0.50	-0.13	-0.44	-0.29	-0.01	-0.02	-0.01	-0.00	39.4787	0.01122	1272	3519.65	<.0001
dummy	1	0.56	-0.03	-0.40	-0.37	-0.01	-0.02	-0.01	-0.00	39.5408	0.01191	1116	3321.17	<.0001
dummy	1	0.63	0.09	-0.33	-0.42	-0.01	-0.02	-0.01	-0.00	39.6021	0.01285	1057	3081.16	<.0001
dummy	1	0.69	0.21	-0.22	-0.42	-0.01	-0.02	-0.01	-0.00	39.6609	0.01410	1068	2813.82	<.0001
dummy	1	0.75	0.34	-0.07	-0.35	-0.01	-0.02	-0.01	-0.00	39.7157	0.01579	1112	2515.78	<.0001
dummy	1	0.81	0.49	0.12	-0.19	-0.01	-0.02	-0.01	-0.00	39.7646	0.01824	1178	2180.29	<.0001
dummy	1	0.88	0.65	0.36	0.07	-0.01	-0.02	-0.01	-0.00	39.8056	0.02190	1318	1817.99	<.0001
dummy	1	0.94	0.82	0.65	0.46	-0.01	-0.02	-0.01	-0.00	39.8365	0.02726	1573	1461.57	<.0001
dummy	1	1.00	1.00	1.00	1.00	-0.01	-0.02	-0.01	-0.00	39.8548	0.03479	1862	1145.70	<.0001

Results for milk protein content (P%) from the period 1-3 days before trait recording

The Mixed Procedure

Model Information

Data Set	WORK.AMS
Dependent Variable	protein
Covariance Structure	Variance Components
Subject Effect	Cow
Estimation Method	REML
Residual Variance Method	Profile
Fixed Effects SE Method	Model-Based
Degrees of Freedom Method	Satterthwaite

Class Level Information

Class	Levels	Values
Lac	5	1 2 3 4 5
Herd	3	A B C
HYS	12	A20223 A20232 B20232 B20233 B20242 B20243 C20232 C20233 C20242 C20243 C20252 C20253
Robot	8	63 64 65 66 67 69 86 87
Cow	790	...
dummy	1	1
Groupsize	3	<40 >=55 40-55
Daytime	3	Nacht Vormittag Nachmittag

Dimensions

Covariance Parameters	5
Columns in X	42
Columns in Z Per Subject	4
Subjects	790
Max Obs Per Subject	1771

Number of Observations

Number of Observations Read	371431
Number of Observations Used	368122
Number of Observations Not Used	3309

Iteration History

Iteration	Evaluations	-2 Res Log Like	Criterion
0	1	-9481.41118670	
1	2	-252532.3935519	0.01191580
2	1	-259986.0588738	0.00864484
3	1	-265409.1786627	0.00598800
4	1	-269175.1461173	0.00401901
5	1	-271705.7461603	0.00261797
6	1	-273353.2427704	0.00164529
7	1	-274385.9669987	0.00098351
8	1	-274999.9587488	0.00054341
9	1	-275335.7821315	0.00026238
10	1	-275494.9126001	0.00009869
11	1	-275552.6710859	0.00002250
12	1	-275565.0078375	0.00000177
13	1	-275565.8959226	0.00000001
14	1	-275565.9026940	0.00000000

Convergence criteria met.

Covariance Parameter Estimates

Cov Parm	Subject	Estimate
lg_thi1_3_1	Cow	0.1180
lg_thi1_3_2	Cow	0.2319
lg_thi1_3_3	Cow	0.1057
lg_thi1_3_4	Cow	0.07717
Residual		0.02657

Fit Statistics

-2 Res Log Likelihood	-275566
AIC (smaller is better)	-275556
AICC (smaller is better)	-275556
BIC (smaller is better)	-275533

Type 1 Tests of Fixed Effects

Effect	Num DF	Den DF	F Value	Pr > F
dummy	1	1953	457921	<.0001
lg_thi1_3_1	1	1428	6.12	0.0135
lg_thi1_3_2	1	971	9.52	0.0021
lg_thi1_3_3	1	917	14.14	0.0002
lg_thi1_3_4	1	817	0.09	0.7630
Lac	4	36E4	879.31	<.0001
lg_dim_1	1	37E4	124.73	<.0001
lg_dim_2	1	37E4	16347.3	<.0001
lg_dim_3	1	37E4	10878.9	<.0001
lg_dim_4	1	37E4	8777.08	<.0001
HYS(Herd)	11	36E4	6768.30	<.0001
Robot(Herd)	7	36E4	777.54	<.0001
Groupsize	2	37E4	9.68	<.0001
Daytime	2	36E4	443.76	<.0001

Type 3 Tests of Fixed Effects

Effect	Num DF	Den DF	F Value	Pr > F
dummy	0	.	.	.
lg_thi1_3_1	1	789	15.36	<.0001
lg_thi1_3_2	1	813	4.68	0.0309
lg_thi1_3_3	1	790	10.23	0.0014
lg_thi1_3_4	1	822	5.58	0.0184
Lac	4	36E4	635.83	<.0001
lg_dim_1	1	37E4	67.25	<.0001
lg_dim_2	1	37E4	19902.7	<.0001
lg_dim_3	1	37E4	10954.3	<.0001
lg_dim_4	1	37E4	9049.72	<.0001
HYS(Herd)	9	37E4	4735.01	<.0001
Robot(Herd)	7	36E4	771.30	<.0001
Groupsize	2	37E4	9.63	<.0001
Daytime	2	36E4	443.76	<.0001

Least Squares Means

Effect	dummy	lg_thi1_3_1	lg_thi1_3_2	lg_thi1_3_3	lg_thi1_3_4	lg_dim_1	lg_dim_2	lg_dim_3	lg_dim_4	Estimate	Standard Error	DF	t Value	Pr > |t|
dummy	1	-1.00	1.00	-1.00	1.00	-0.01	-0.03	-0.01	-0.00	3.2785	0.02823	2826	116.13	<.0001
dummy	1	-0.94	0.82	-0.65	0.46	-0.01	-0.03	-0.01	-0.00	3.3091	0.02163	2405	152.98	<.0001
dummy	1	-0.88	0.65	-0.36	0.07	-0.01	-0.03	-0.01	-0.00	3.3335	0.01689	1970	197.37	<.0001
dummy	1	-0.81	0.49	-0.12	-0.19	-0.01	-0.03	-0.01	-0.00	3.3522	0.01372	1712	244.39	<.0001
dummy	1	-0.75	0.34	0.07	-0.35	-0.01	-0.03	-0.01	-0.00	3.3660	0.01175	1625	286.50	<.0001
dummy	1	-0.69	0.21	0.22	-0.42	-0.01	-0.03	-0.01	-0.00	3.3756	0.01058	1632	319.01	<.0001
dummy	1	-0.63	0.09	0.33	-0.42	-0.01	-0.03	-0.01	-0.00	3.3815	0.009869	1706	342.65	<.0001
dummy	1	-0.56	-0.03	0.40	-0.37	-0.01	-0.03	-0.01	-0.00	3.3843	0.009392	1862	360.32	<.0001
dummy	1	-0.50	-0.13	0.44	-0.29	-0.01	-0.03	-0.01	-0.00	3.3844	0.009057	2088	373.68	<.0001
dummy	1	-0.44	-0.21	0.45	-0.18	-0.01	-0.03	-0.01	-0.00	3.3824	0.008843	2285	382.50	<.0001
dummy	1	-0.38	-0.29	0.43	-0.07	-0.01	-0.03	-0.01	-0.00	3.3788	0.008756	2272	385.88	<.0001
dummy	1	-0.31	-0.35	0.39	0.05	-0.01	-0.03	-0.01	-0.00	3.3738	0.008793	2000	383.71	<.0001
dummy	1	-0.25	-0.41	0.34	0.16	-0.01	-0.03	-0.01	-0.00	3.3679	0.008925	1645	377.35	<.0001
dummy	1	-0.19	-0.45	0.26	0.25	-0.01	-0.03	-0.01	-0.00	3.3614	0.009106	1361	369.14	<.0001
dummy	1	-0.13	-0.48	0.18	0.32	-0.01	-0.03	-0.01	-0.00	3.3546	0.009280	1177	361.48	<.0001
dummy	1	-0.06	-0.49	0.09	0.36	-0.01	-0.03	-0.01	-0.00	3.3476	0.009399	1077	356.18	<.0001
dummy	1	0.00	-0.50	0.00	0.38	-0.01	-0.03	-0.01	-0.00	3.3408	0.009428	1041	354.34	<.0001
dummy	1	0.06	-0.49	-0.09	0.36	-0.01	-0.03	-0.01	-0.00	3.3343	0.009356	1062	356.38	<.0001
dummy	1	0.13	-0.48	-0.18	0.32	-0.01	-0.03	-0.01	-0.00	3.3281	0.009191	1145	362.12	<.0001
dummy	1	0.19	-0.45	-0.26	0.25	-0.01	-0.03	-0.01	-0.00	3.3224	0.008964	1304	370.64	<.0001
dummy	1	0.25	-0.41	-0.34	0.16	-0.01	-0.03	-0.01	-0.00	3.3173	0.008726	1555	380.15	<.0001

dummy	1	0.31	-0.35	-0.39	0.05	-0.01	-0.03	-0.01	-0.00	3.3126	0.008539	1869	387.95	<.0001
dummy	1	0.38	-0.29	-0.43	-0.07	-0.01	-0.03	-0.01	-0.00	3.3085	0.008460	2102	391.06	<.0001
dummy	1	0.44	-0.21	-0.45	-0.18	-0.01	-0.03	-0.01	-0.00	3.3048	0.008531	2090	387.37	<.0001
dummy	1	0.50	-0.13	-0.44	-0.29	-0.01	-0.03	-0.01	-0.00	3.3014	0.008763	1902	376.72	<.0001
dummy	1	0.56	-0.03	-0.40	-0.37	-0.01	-0.03	-0.01	-0.00	3.2981	0.009149	1714	360.50	<.0001
dummy	1	0.63	0.09	-0.33	-0.42	-0.01	-0.03	-0.01	-0.00	3.2948	0.009691	1603	339.98	<.0001
dummy	1	0.69	0.21	-0.22	-0.42	-0.01	-0.03	-0.01	-0.00	3.2913	0.01045	1559	314.88	<.0001
dummy	1	0.75	0.34	-0.07	-0.35	-0.01	-0.03	-0.01	-0.00	3.2872	0.01160	1546	283.36	<.0001
dummy	1	0.81	0.49	0.12	-0.19	-0.01	-0.03	-0.01	-0.00	3.2822	0.01343	1583	244.40	<.0001
dummy	1	0.88	0.65	0.36	0.07	-0.01	-0.03	-0.01	-0.00	3.2760	0.01632	1760	200.79	<.0001
dummy	1	0.94	0.82	0.65	0.46	-0.01	-0.03	-0.01	-0.00	3.2683	0.02063	2119	158.39	<.0001
dummy	1	1.00	1.00	1.00	1.00	-0.01	-0.03	-0.01	-0.00	3.2585	0.02670	2517	122.02	<.0001
